# A wide range of South American inselberg floras reveal cohesive biome patterns

**DOI:** 10.3389/fpls.2022.928577

**Published:** 2022-09-29

**Authors:** Rafael Gomes Barbosa-Silva, Caroline O. Andrino, Luísa Azevedo, Luísa Lucresia, Juliana Lovo, Alice L. Hiura, Pedro L. Viana, Tereza C. Giannini, Daniela Cristina Zappi

**Affiliations:** ^1^ Biodiversity and Ecosystem Services, Instituto Tecnológico Vale Desenvolvimento Sustentável, Belém, Brazil; ^2^ Coordenação Botânica, Museu Paraense Emílio Goeldi, Belém, Brazil; ^3^ Departamento de Genética, Ecologia e Evolução, Universidade Federal de Minas Gerais, Belo Horizonte, Brazil; ^4^ Instituto de Biociências, Departamento de Botânica, Universidade de São Paulo, São Paulo, Brazil; ^5^ Departamento de Sistemática e Ecologia/Programa de Pós-Graduação em Ecologia, e Monitoramento Ambiental, Universidade Federal da Paraíba, João Pessoa, Brazil; ^6^ Programa de Pós-Graduação em Zoologia, Instituto de Ciência Biológicas, Universidade Federal do Pará, Belém, Brazil; ^7^ Programa de Pós-Graduação em Botânica, Instituto de Ciências Biológicas, Universidade de Brasília, Distrito Federal, Brazil

**Keywords:** Amazon, Atlantic forest, Caatinga, floristics, granitic outcrops, neotropical flora, phylogenetic comparisons, similarity analyses

## Abstract

Inselbergs are azonal formations found scattered in different biomes globally. The first floristic list focusing on an inselberg in the Brazilian Amazon is presented here. We aimed to investigate floristic and phylogenetic connections among Neotropical inselbergs and analyze whether environmental variables act as a filter of plant lineages. We used a database compiled from 50 sites spanning three main Neotropical biomes (Amazon, 11 sites, Atlantic Forest, 14 sites, and Caatinga, 25 sites) comprising 2270 Angiosperm species. Our data highlight the vastly different inselberg flora found in each biome. The inselberg floras of the Atlantic Forest and Caatinga show closer phylogenetic ties than those seen in the other biome pairs. The phylogenetic lineages found in all three biomes are also strongly divergent, even within plant families. The dissimilarity between biomes suggests that distinct biogeographical histories might have unfolded even under comparable environmental filtering. Our data suggest that the inselberg flora is more related to the biome where it is located than to other factors, even when the microclimatic conditions in the outcrops differ strongly from those of the surrounding matrix. Relative to the other biomes, the flora of the Caatinga inselbergs has the highest level of species turnover. There is a possibility that plants colonized these rather distant inselbergs even when they were found under very different climatic conditions than those in the Amazonian and Atlantic Forest biomes. It is worth noting that none of the studied inselbergs found in the Caatinga biome is protected. In view of the uniqueness and drought-resilient lineages present in each group of inselbergs, along with their vulnerability to destruction or disturbance and their strong connection with water availability, we stress the need to protect this ecosystem not only to conserve plants potentially useful for ecological restoration but also to preserve the balance of this ecosystem and its connections.

## Introduction

Inselbergs are isolated havens for dryland flora scattered over different biomes and ecoregions globally (Barthlott et al., 1996). Geomorphologically, these outcrops consist of extremely hard gneiss and/or granite rocks that survive the process of erosion around them, forming rock surfaces with low water holding capacity and, because of their dark color, often overheating under the inclement tropical sun (Kluge and Büdel, 1996). Inselbergs can be considered azonal, as outcrops are located within perhumid tropical American regions, such as Amazonia and the Atlantic Forest in South America, being home to typically xeric vegetation. In such outcrops plants establish on very shallow soil, under wide daily temperature fluctuations and with limited water availability (Ibisch et al., 1995; Prance, 1996; Sarthou and Villiers, 1998; de Paula et al., 2015; de Paula et al., 2020). Species thriving in microhabitats with shallow soil are usually stress-tolerant, therefore concentrating around the S strategy of the Grime triangle (de Paula et al., 2015). Stress-tolerant adaptations displayed by tropical American inselberg flora include plant mat formation, desiccation tolerance, deciduous leaves, photosynthetic stems, water reserves in succulent organs, bromeliad tanks, orchid pseudobulbs, and the presence of spines or thorns (Barthlott and Porembski, 1996; Gröger, 2000; de Paula et al., 2020). Plants growing on these outcrops have been used as models for gene-flow studies, and the populations have presented strong geographical structure and long-term persistence in the sites (Barbará et al., 2009; Millar et al., 2013; Tapper et al., 2014; Hmeljevski et al., 2017; Mota et al., 2020). Until now, there has been no angiosperm survey for inselbergs in the Brazilian Amazon, even though there are published lists for neighboring countries (Raghoenandan, 2000; Sarthou et al., 2003; Gutiérrez et al., 2007). Inselbergs in Venezuela occupy the edge of the Guayana Shield and are located on the border between different phytogeographical regions (Gröger, 2000). Likewise, in Brazil, these formations are found on the Guayana Shield and the eroded edges of the Central Brazilian Shield. However, they are scattered within and at the borders of different biomes, with the Atlantic Forest being home to the iconic Sugarloaf Mountains (de Paula et al., 2020).

Despite the recent progress in listing all plant species from the Amazonian lowland rainforest (Cardoso et al., 2017), other vegetation types within the region have not yet been examined in detail, and the beta diversity of the region is still poorly understood (Milliken et al., 2010; Zappi et al., 2011; Costa et al., 2020). Since Amazonia fulfills an important role in (1) maintaining the rain regime, (2) lowering the temperature throughout the continent (Lovejoy and Nobre, 2019) and (3) indirectly preserves public health (Ellwanger et al., 2020), it is paramount to understand and preserve all species that maintain the complex web of interactions found within all its ecosystems. Moreover, it is important to be aware of which species inhabit drier areas within Amazonia and other forest biomes, as they may be key to future restoration projects in light of climate change (Miranda et al., 2021). On the other hand, it is possible that some of these species behave as edaphic specialists (Corlett and Tomlinson, 2020) that may be pushed past their tolerance threshold as temperatures on Earth increase, being vulnerable to climate change.

Floristic inventories are an important source of biodiversity data, both at the local and regional levels. Such lists represent fundamental contributions toward large databases and provide baseline information to improve our knowledge regarding biodiversity in tropical America (Queiroz et al., 2006; Mendonça et al., 2008; Forzza et al., 2010; Cardoso et al., 2017; Funk, 2018; Mendieta-Leiva et al., 2020), having practical use for habitat restoration and climatic change predictions (Roloff et al., 2009; Zappi et al., 2022). Complete floristic datasets that include all life forms also allow us to perform a wide array of analyses that may shed light on the different elements of the landscape that are responsible for high beta diversity (Zappi et al., 2017; Andrino et al., 2020; Devecchi et al., 2020). Here, we present the first complete angiosperm list focusing on an inselberg flora in Brazilian Amazonia. To perform floristic comparisons, we compiled the majority of the floristic lists of inselbergs available for South America where all life forms were considered, preparing the most complete tropical American inselberg species list. We investigated whether the floristic relationships between inselbergs have greater affinity across or within the tropical American biomes recognized by Antonelli et al. (2018). We analysed whether the inselberg environment filters (1) similar lineages and (2) specific environmental aspects related to species turnover in these habitats. Thus, we aimed to test whether biomes represent boundaries for floristic transitions within this specific habitat and whether they impose different adaptive challenges to the different floristic lineages.

## Methods

### New floristic list for the amazon

The Pedra da Harpia (PHR) is an inselberg located in the Brazilian state of Pará in eastern Amazonia, located within two protected areas, the Carajás National Forest and the Campos Ferruginosos National Park, in the municipality of Canaã dos Carajás. Immersed in a matrix of open ombrophilous and semideciduous forest, the PHR granitic outcrop reaches an altitude of 590 m a.s.l. (-6.284003, -50.336854), forming a 60-meter flat-topped wall descending to a 45-degree slope. Botanical specimens from the PHR housed in the Herbarium of the Museu Paraense Emílio Goeldi (MG) and in the Herbarium of the Federal University of Minas Gerais (BHCB) (Thiers, 2021) collected prior to 2017 were compiled into a database. In addition, nine expeditions were carried out between 2017 and 2020 (February 2017, May 2017, June 2017, December 2018, May 2019, July 2019, October 2019, November 2019, February 2020 and April 2022), aiming to collect fertile material of vascular species throughout the seasons. The collection method followed Filgueiras et al. (1994), with random walks covering the accessible parts of the inselberg. Voucher specimens were deposited at MG. One voucher per taxon is presented in the floristic list provided (S2). Species names were assigned according with the Flora do Brasil online resource (Flora do Brasil, 2020), family delimitation followed the Angiosperm Phylogeny Group IV (APG IV) system (The Angiosperm Phylogeny Group, 2016) and author abbreviations followed the International Plant Names Index (IPNI) (IPNI, 2022).

### Inselberg dataset

Fourty nine floristic lists of South American granitic inselbergs were compiled from 33 published papers (**Supplementary Tables S1**, **2**) along with the new checklist for the Amazonian inselberg PHR (**Supplementary Table S3**). The abbreviations corresponding to each area, are specified in **Supplementary Table S1**. Our analysis took into consideration sites with more than 25 species found below 1000 m a.s.l., preventing the inclusion of the Atlantic Campos de Altitude vegetation (de Paula et al., 2016). For the analysis, we focused only on Angiosperms. We are aware that the sampling performed to compile the lists may have included species from the surrounding forest matrix as well as the outcrop species (especially in a few Atlantic Forest sites). As these lists mostly did not include microhabitat data, we did not attempt to separate the vegetation types in our analyses.

The lists were transcribed according to the original manuscripts, and the names were checked against data from the Flora do Brasil and The Plant List using the ‘Flora’ package in R (Carvalho, 2022). All synonyms were updated, and when possible, we examined voucher specimens when identities appeared as incompletely named, invalid or as not occurring in Brazil. In several instances, specimens were reidentified, and the name record was updated. Specimens named to only the generic level (**Supplementary Table S4**) were excluded, as well as morphospecies included in some checklists and exotic invasive species. Specimens identified with *cf.* were grouped together with the species with which it was compared, while specimens annotated with *aff.* were considered different from the species name (possible new species or records).

### Biogeographic patterns of neotropical inselbergs

Inselbergs were grouped according to their occurrence in tropical American biomes following Antonelli et al. (2018). We explored the floristic similarity patterns between inselbergs by performing nonmetric multidimensional scaling (NMDS) and unweighted pair group method with arithmetic mean (UPGMA) using a presence-absence matrix with the ‘Vegan’ package (Oksanen et al., 2010) and the ‘ggplot2’ package (Wickham, 2009) in R software.

Floristic links among biomes and between sites within each biome were investigated and computed in chord diagrams with the ‘Circlize’ package in R software (Gu et al., 2014). Species richness and the number of shared species among biomes were estimated with incidence data using Chao2 with the ‘SpadeR’ package in R software (Chao et al., 2016).

For each inselberg, we also calculated the proportion of exclusive species and the proportion of species shared with other areas, creating an asymmetric matrix. These proportions were computed graphically and combined with the UPGMA results in a heatmap with the ‘ComplexHeatmap’ package in R software (Gu et al., 2016). The phylogenetic structure of the inselberg plant communities, including our species list, was constructed with the phylomatic tool (Webb and Donoghue, 2005) in Phylocom version 4.1. (Webb et al., 2008) using megatree R20160415.new (Gastauer and Meira-Neto, 2016). The estimated ages for each node followed (Bell et al., 2010). Subsequently, iTOL (Letunic and Bork, 2016) was used to visualize the inselberg megatree, highlight selected plant families and show species occurrences within the three Neotropical biomes (Amazon, Atlantic Forest, and Caatinga). In the megatree, the estimated age of each lineage is proportionally represented by the branch length.

### Influence of large-scale environmental variables

Included areas were georeferenced by checking the GPS location provided in the study sites and comparing the coordinates with high-quality maps. Generalized dissimilarity modeling (GDM) was used to investigate the role of climatic factors and geographical distance on species composition across the different inselbergs (Ferrier et al., 2007). GDM uses maximum likelihood and I-splines to analyse and predict species turnover as a function of environmental conditions and geographic distance (Ferrier et al., 2007). Environmental predictors were obtained from WorldClim (Fick and Hijmans, 2017) as raster files and their medians were compiled in QGIS software (version 3.6.2). Predictors with greater explanatory power were selected through backward elimination and ranked according to significance in a preliminary model. Since GDM combines elements of generalized linear modeling and matrix regression, the resultant model could be sensitive to correlated explanatory variables (Leathwick et al., 2006). Therefore, predictors with a Pearson correlation coefficient of 0.7 or higher were removed, and selection between correlated variables prioritized predictors with the highest relevance in the model, maintaining variables with greater explanatory power. This procedure led to the exclusion of 10 predictors: precipitation of the wettest month, precipitation of the driest month, precipitation of the wettest quarter, precipitation of the driest quarter and precipitation of the coldest quarter due to high correlation with annual precipitation; and annual mean temperature, isothermality, maximum temperature of the warmest month, mean temperature of the driest quarter and mean temperature of the coldest quarter due to correlation with minimum temperature of the coldest month. A new model was fitted after removing the correlated predictors, and since the explanatory power of the model was not affected, the reduced model was kept. The statistical significance of the selected model and included climatic predictors were tested with 999 permutations (α=0.05). Statistical analyses and graphing were performed with the ‘gdm’ package in R software. It is important to mention that microclimate data for the studied inselbergs are unavailable as yet, and would be paramount to refine the comparisons.

Statistical analyses and graphing were performed with the ‘gdm’ package in R software (Fitzpatrick et al., 2021). All scripts are supplied in **Supplementary information (S5)**.To map the sampled inselbergs relative to protected areas, we used from the World Database on Protected Areas (2016).

## Results

The new Amazonian floristic list of the PHR contributed 125 species, with *Fabaceae* (or *Leguminosae*) (13 spp.) being the richest family in this inselberg, followed by *Euphorbiaceae* (8 spp.) and *Malvaceae* (7 spp.). Seventy-two species (60.5%) on this new list do not occur on any of the other inselbergs included in this study, including the recently described *Alophia graniticola* A. Gil, an endemic species exclusive to the PHR (Gil et al., 2021). For a detailed list of species, see the electronic **Supplementary information (S3)**.

**Figure 1:**
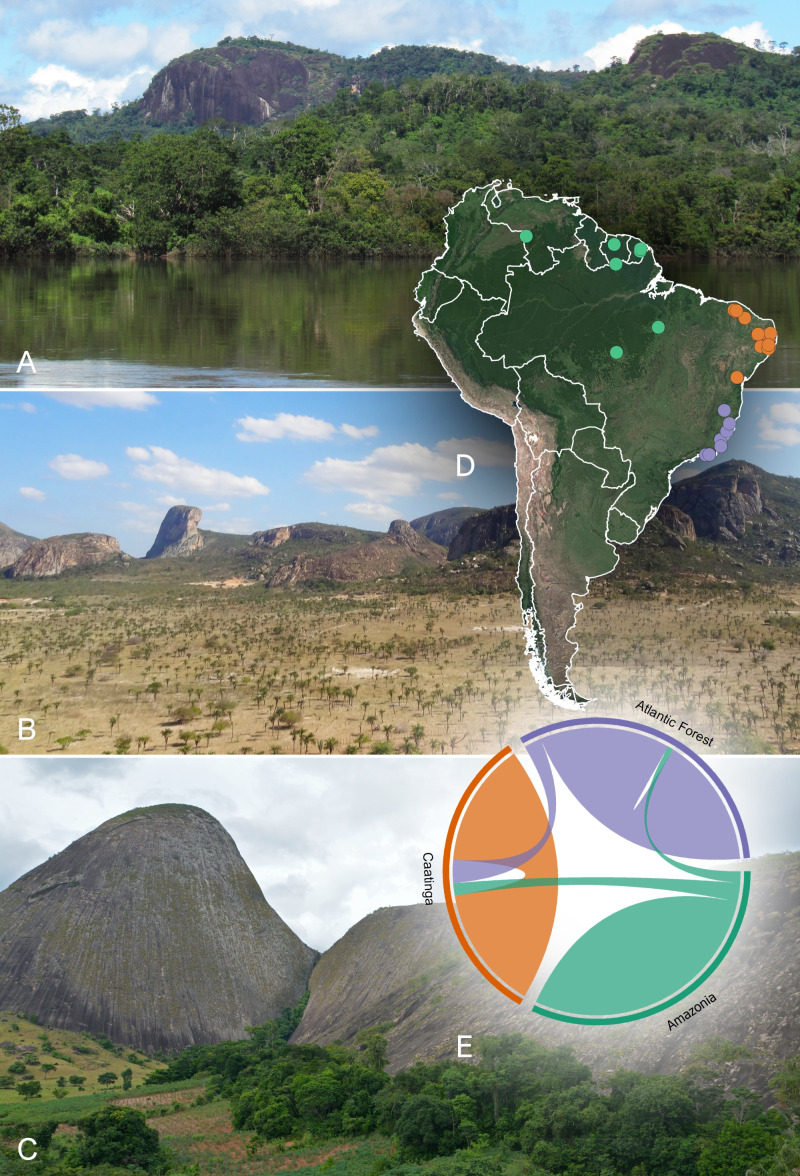
Inselbergs located in the three Neotropical biomes analysed: Amazonian **(A)** Serra Grande, Cantá municipality; Caatinga **(B)** Itatim municipality; and Atlantic Forest **(C)** Pedra do Caladão, Carlos Chagas municipality, Minas Gerais state. **(D)** Distribution map of the 50 inselbergs in South American sites used in the analyses with their classification by Neotropical biome. The green markers indicate the inselbergs located in the Amazonian biome, orange markers indicate those located in the Caatinga biome, and mauve markers indicate those in the Atlantic Forest biome. **(E)** Overlap in species composition between inselbergs in the Amazonian, Caatinga and Atlantic Forest biomes. Photos **a-b** RGBS; **c** LA.

**Figure 2:**
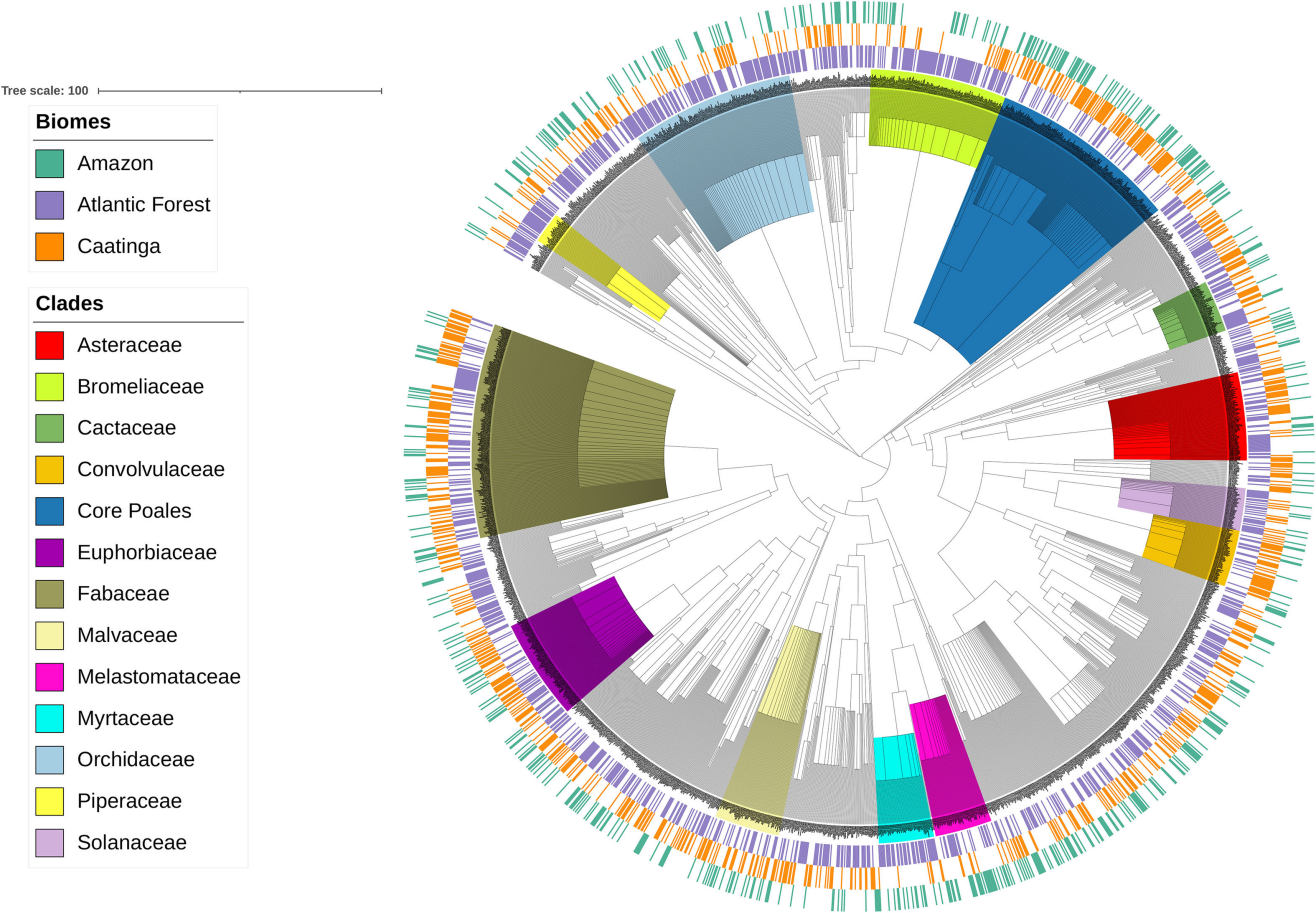
The inselberg megatree indicates the different biomes of 2193 species: the outer ring (orange) represents the Caatinga inselbergs, the middle ring (mauve), the Atlantic Forest inselbergs, and the inner ring (green) the Amazonian inselbergs. The plant groups mentioned in the results and discussion are highlighted.

This new list (PHR) was merged with 49 already published floristic lists, comprising 50 neotropical inselbergs (S1, S2). Our analyses yielded 2193 angiosperm species distributed in 140 families and 806 genera (**Table S4**), totaling 4397 occurrences spanning over three biomes: Amazonia, Caatinga and Atlantic Forest (**Figure 1**). The 11 Amazon inselbergs sampled yielded 540 species (out of 776 records); 1121 species (out of 1782 collections) were recorded for the 14 inselbergs sampled in the Atlantic Forest, and 785 species (1837 collections) were recorded for the 25 inselbergs sampled in the Caatinga. The phylogenetic tree (**Figure 2**) shows very little correspondence between the lineages found in each biome, with a strong association of certain exclusive clades in the Caatinga inselbergs and many other clades associated with the Atlantic Forest. Proportionally fewer clades can be observed in Amazonian inselbergs.

### Biogeographic patterns of Neotropical inselbergs

**Figure 3:**
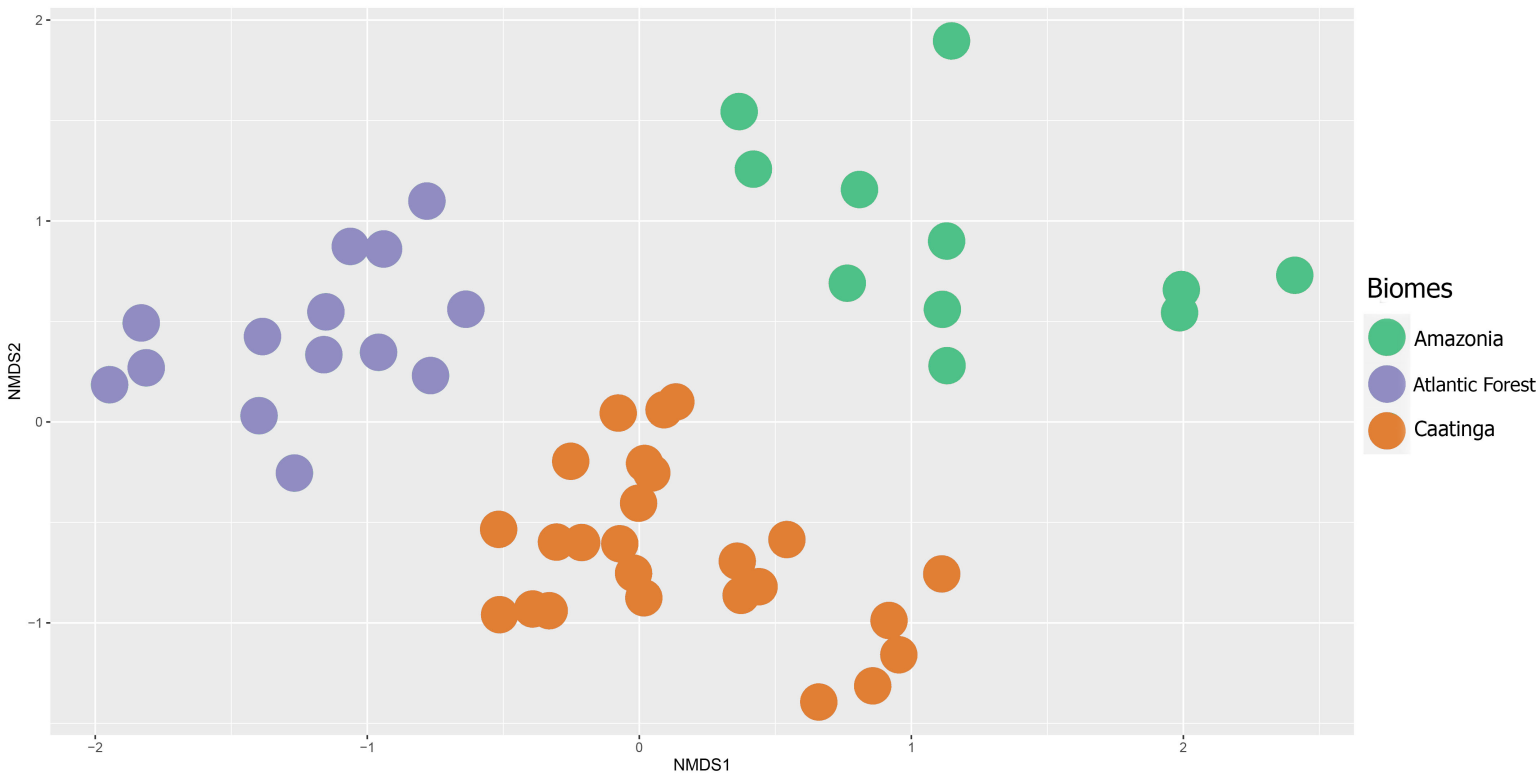
Ordination of 50 inselbergs in South America inferred from nonmetric multidimensional scaling of their species composition. Colors indicate the *a priori* classification into the main Neotropical biomes. Orange = Caatinga; Mauve = Atlantic Forest; Green = Amazon.

**Figure 4:**
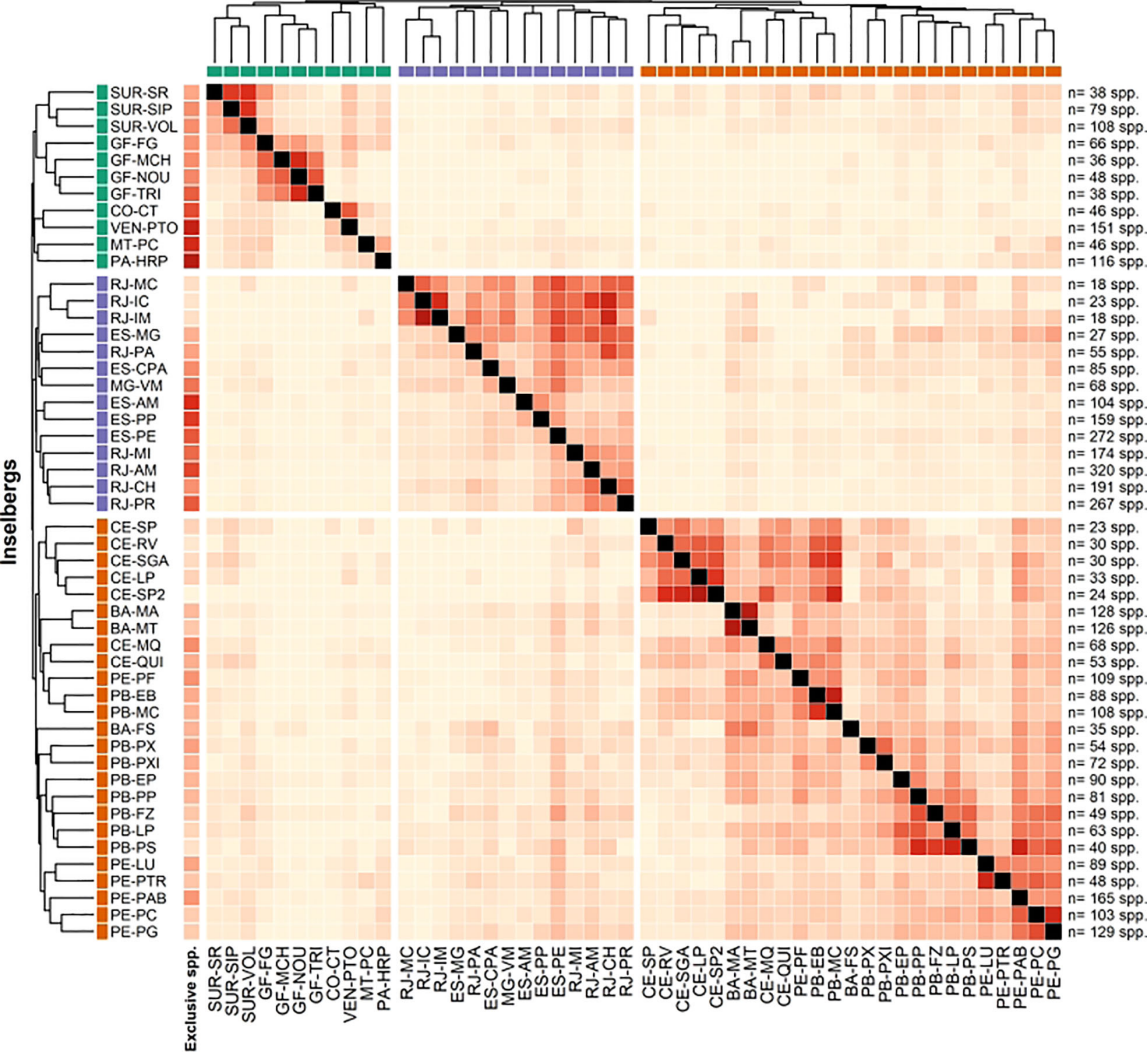
Heatmap of similarity between the inselberg floristics datasets. Dendrograms were plotted using the unweighted-pair-group method with arithmetic mean (UPGMA). The colors indicate the proportion of shared species, ranging from yellow (low values, less similarity) to red (high values, higher similarity).

The biogeographical analyses revealed little similarity among the sampled inselbergs, organizing them according to the Neotropical biomes where they occur, namely, Atlantic Forest, Caatinga and Amazonia. The distribution of inselbergs in ordination space yielded by NMDS (stress = 0.2331; **Figure 3**) revealed three clear, cohesive biome groups corresponding to the Atlantic Forest, Caatinga and Amazonian inselbergs (ANOSIM R = 0.8421; p = 0.001), with the Amazonian inselbergs separated by a diagonal line from the Atlantic Forest and Caatinga inselbergs. Of these three groups, the Amazonian sites appear more scattered, indicating a higher beta diversity, while the Atlantic Forest and Caatinga sites appear more condensed, suggesting that they are more similar in species composition and have lower beta diversity than the Amazonian sites (**Figure 1E**). The Amazonian inselbergs shared fewer species (19.5%) with the other biomes, while areas in the Caatinga and Atlantic Forest biomes were more similar in terms of shared species. The UPGMA showed that proportionally, the inselbergs in the Caatinga biome shared more species with the other two biomes (25.5%), while the inselbergs in the Atlantic Forest biome shared fewer species with the Amazonian and Caatinga biomes (16%) (**Figure 4**; Additional chord diagrams can be found under **Supplementary information (S6)**).

Estimated richness of inselbergs within studied biomes predicted a higher diversity of inselbergs within the Atlantic Forest (2,496 spp.), followed by Amazonia (1,350 spp.) and Caatinga (1,189 spp.), indicating a more profound subsample in the Amazon – where only 40% of estimated inselberg species were observed. The estimated number of shared species among inselbergs in distinct biomes predicted a higher amount of shared spp. but yielded similar patterns regarding floristic links among biomes: 179 spp. shared between the Amazon and Atlantic Forest (with 49 spp. observed); 234 spp. shared between Amazon and Caatinga (with 80 spp. observed); and 330 spp. shared among Atlantic Forest and Caatinga (with 149 spp. observed) (see chord diagrams under **Supplementary information S6**).

In terms of plant families, *Bromeliaceae, Orchidaceae, Euphorbiaceae* and *Fabaceae* (*Leguminosae*) are the most commonly represented families in the inselbergs for all three ecoregions. The *Orchidaceae* are the most species-rich family among the Atlantic Forest inselbergs, while *Fabaceae (Leguminosae)* and *Poaceae* are the richest families in the Caatinga and Amazonian biomes, respectively. Asteraceae is among the 10 richest families shared between the Atlantic Forest and the Caatinga biomes, while *Poaceae, Cyperaceae*, and *Malvaceae* are the richest families shared between the Amazonian and Caatinga biomes. Only one family, Bromeliaceae, is among the richest families shared between the Amazonian and Atlantic Forest biomes, although it is not one of the richest families in the Caatinga biome. Convolvulaceae is among the richest families only for the Caatinga inselbergs, while Melastomataceae is among the richest families only in the Amazonian inselbergs. Likewise, *Cactaceae, Myrtaceae*, and *Solanaceae* are among the richest families only in the Atlantic Forest inselbergs.

The megatree showed that eighteen families were recorded exclusively in the Atlantic Forest biome, namely, *Achariaceae, Aquifoliaceae, Araliaceae, Asparagaceae, Balanophoraceae, Calophyllaceae, Campanulaceae, Cannaceae, Dilleniaceae, Elaeocarpaceae, Erythroxylaceae, Monimiaceae, Oleaceae, Peraceae, Picramniaceae, Simaroubaceae, Tropaeolaceae* and *Ulmaceae*. The eight families exclusive to the Caatinga biome were *Alismataceae, Connaraceae, Hypoxidaceae, Pontederiaceae, Ranunculaceae, Vochysiaceae, Zingiberaceae* and *Zygophyllaceae*. The ten families exclusive to the Amazonian rainforest were *Haemodoraceae, Strelitziaceae, Cyclanthaceae, Pentaphylacaceae, Caricaceae, Caryophyllaceae, Dichapetalaceae, Opiliaceae, Linderniaceae* and *Siparunaceae*.

### Influence of large-scale environmental variables

**Figure 5:**
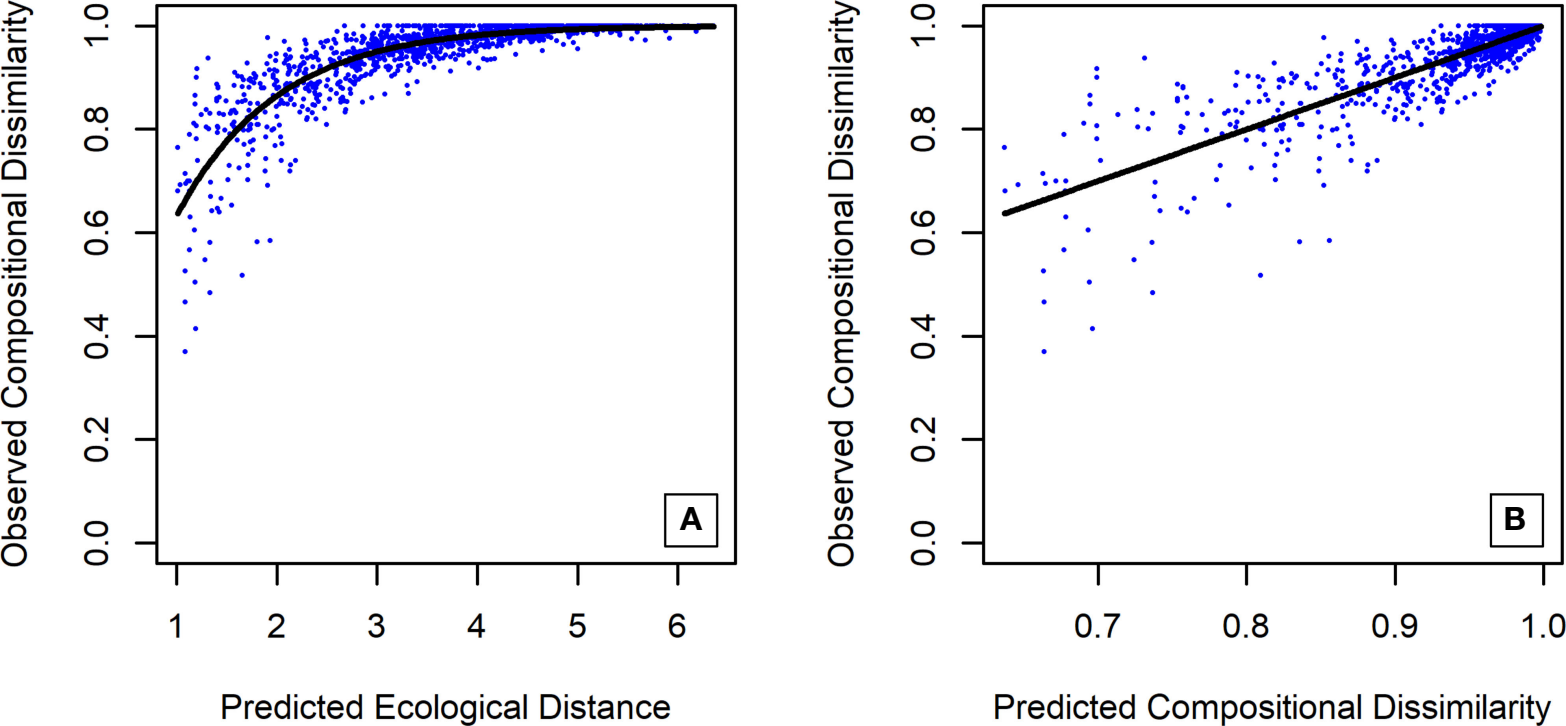
Diagnostic plots from generalized dissimilarity modeling (GDM) of observed compositional dissimilarity against predicted ecological distance **(A)** and predicted compositional dissimilarity **(B)**.

**Figure 6:**
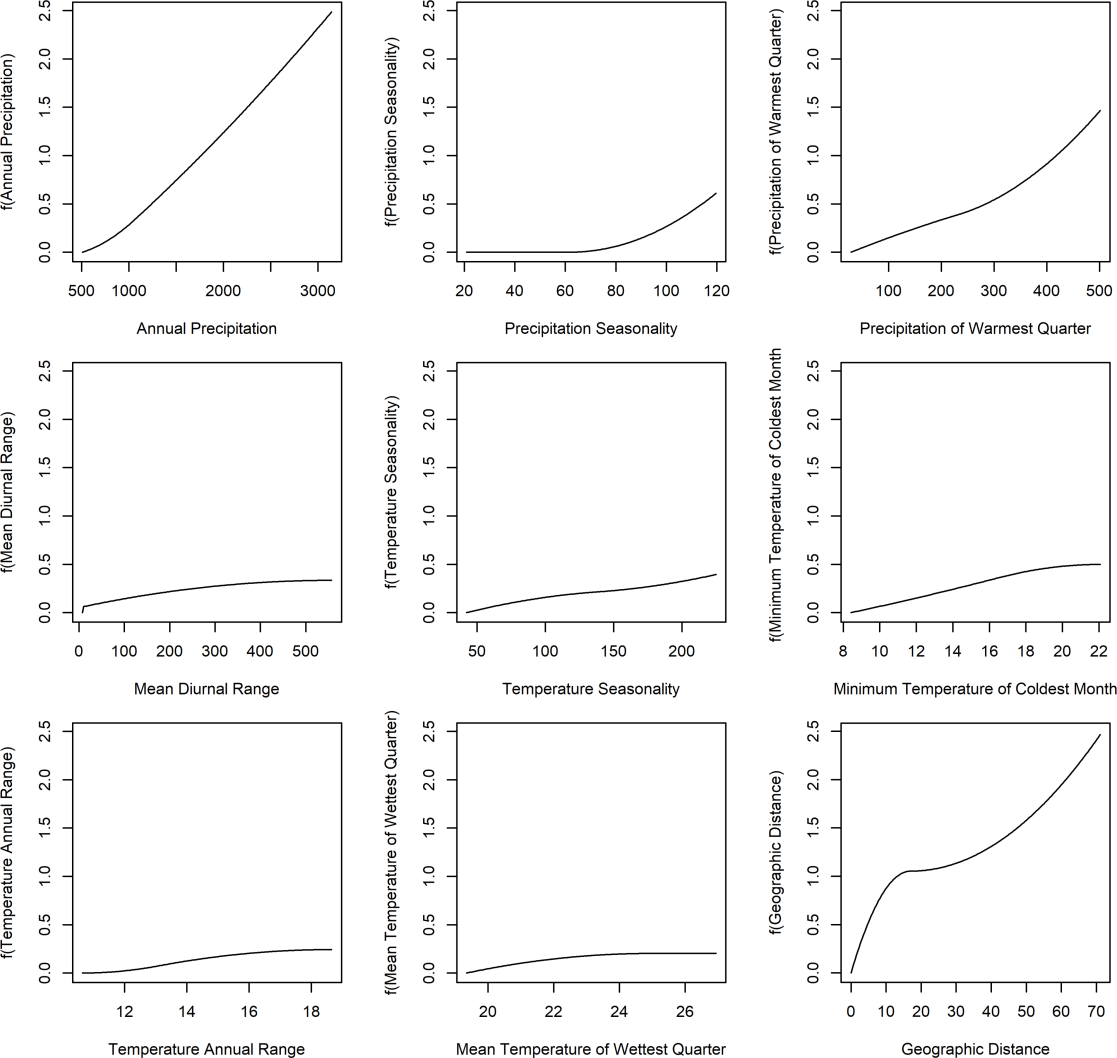
Fitted I splines from the generalized dissimilarity models for the nine environmental predictors associated with species turnover in inselbergs. The horizontal axes correspond to the variation in a given predictor, and vertical axes represent the species turnover suggested by dissimilarity patterns.

The GDM explained 76% of the variance found within the dataset, with 27.58% attributed solely to geographical distance and 48.42% explained by a combination of eight climatic predictors (**Table 1**). Three environmental variables associated with precipitation accounted for 44.81% of the explained variance and were the only climatic predictors with statistical significance: annual precipitation (31.85%), precipitation of warmest quarter (7.85%) and precipitation seasonality (5.10%). Climatic predictors associated with temperature were selected by the model but explained only c. 3.5% of the total variation: minimum temperature of coldest month (0.96%), mean temperature of wettest quarter (0.94%), mean diurnal range (0.81%), temperature seasonality (0.53%) and temperature annual range (0.36%). The mean temperature of the warmest quarter and elevation were not relevant for species turnover and, although included in the analysis, were dropped by backward elimination. Plots of observed dissimilarity in species composition against predicted ecological distance and predicted compositional dissimilarity are presented in **Figure 5**, and the fitted I-splines to each of the relevant predictors are presented in **Figure 6**.

**Table 1:** Predictors included in the generalized dissimilarity models, their proportional relevance for species turnover and statistical significance.

Environmental predictor	Relevance (%)	Significance (p)
Annual precipitation	31.85%	0.00
Geographic distance	27.58%	0.00
Precipitation of warmest quarter	7.85%	0.00
Precipitation seasonality	5.10%	0.00
Minimum temperature of coldest month	0.96%	0.19
Mean temperature of wettest quarter	0.94%	0.16
Mean diurnal range	0.81%	0.24
Temperature seasonality	0.53%	0.24
Temperature annual range	0.36%	0.38
Not explained	24.00%	

### Mapping Neotropical inselbergs onto protected areas

Only sixteen out of the 50 inselbergs (32%) included in the analysis are protected, of which nine are found in Amazonia, each one in a different protected area. Seven are in the Atlantic Forest, corresponding to only six protected areas. None of the studied inselbergs in the Caatinga are found within protected areas. Further details about the locations of the sampled inselbergs in protected areas can be found in **Supplementary information S6**.

## Discussion

Neotropical inselbergs have formed cohesive groups coinciding with the three biomes involved (Amazonia, Caatinga and Atlantic Forest), while little similarity was found between inselbergs across biomes at the species level. There is a high level of biotic interchange between the inselbergs found in the Caatinga, as opposed to the Amazon, which has the highest proportion of exclusive species. Our inselberg megatree also revealed little correspondence between lineages for the three groups. The GDM corroborated that the matrix, in which inselbergs are located, is potentially relevant to species turnover while the significant precipitation differences among the biomes is highlighted.

### Contrasting floras in each biome

Among the ten richest families in the Amazonian lowland forest (Cardoso et al., 2017), six are also the richest in the Amazonian inselbergs. For the Atlantic Forest, seven of the ten richest inselberg families coincide with those in the Atlantic Forest matrix as a whole (BFG, 2015). In the case of the Caatinga biome, this number is even higher, with nine out of the ten richest inselberg families also being the richest in the biome (BFG, 2015). This pattern was not observed for other Amazon rupicolous ecosystems on different rock substrates (non-granitic), such as Pantepui in the Guayana Shield (Berry and Riina, 2005) or the *canga* of Carajás in the eastern Amazon (Mota et al., 2018), where family richness was found to contrast that in the surrounding biome.

The strong floristic clustering of the inselbergs in each biome, associated with the scant network of shared species between the Amazonian, Caatinga, and Atlantic Forest biomes, suggests that the vegetation matrix plays an important role in the biota of these outcrops (de Queiroz et al., 2017). To better understand this role, it is paramount to compare the inselberg plant community with its surrounding matrix. As seen in French Guiana, inselberg forests have a significantly similar plant species composition in relation to their matrix (Sarthou et al., 2003), but this similarity may be compromised if considering only rupicolous plants (Esgario et al., 2009). Additionally, to further refine our understanding, it is fundamental that floristic lists specify the microhabitat in which the species were recorded. This would allow the investigation of inselberg insularity and the permeability of their matrix. Due to the region´s high biodiversity and lack of adequate sampling, we do not yet know which species are endemic to inselberg ecosystems in the Neotropics and therefore are unable to determine whether the outcrops function as islands for at least some plant groups and whether the surrounding biome functions (or functions) as a “mainland”, isolating the outcrop´s biota (Itescu, 2019).

Herein, we report a high level of species turnover among all the inselbergs found in the Caatinga biome (**Figure 4**), probably related to the xeric nature of this biome (precipitation between 300 and 800 mm/year), a condition also found on the inselbergs. The permeability of an arid matrix is higher than that of a humid one (McGann, 2002), justifying a greater species turnover between Caatinga inselbergs. Within South America and considering the three sampled biomes, the inselbergs with the highest sharing of angiosperm species are located in the Caatinga biome, encompassing 25% of the species found in the other biomes. These results show the complexity of the xeric rupicolous vegetation of the inselbergs. Two inselbergs from northeastern Brazil (PE-LU and PE-PTR) are found in the inland Atlantic Forest biome (precipitation between 750 and 1200 mm/year), near the border of the Caatinga, and were grouped with the latter biome, evidencing the strong species links found between the xeric conditions of the inselbergs and this biome in particular. The 787 species in the Caatinga inselbergs, considering the much smaller size of this biome’s flora when compared with those of the Atlantic Forest and Amazonian biomes (Flora do Brasil, 2020), highlights a considerable number of plants that are highly adapted to dry and extreme conditions.

Even considering the relatively small geographic distance between the Atlantic Forest inselbergs studied, the total species number (1123) and uniqueness of each inselberg are quite remarkable, evidencing the biome as a recognized global hotspot (Myers et al., 2000). The set of different plant families that appeared exclusively in the Atlantic Forest inselbergs also reflects this diversity. In terms of species count, the Atlantic Forest biome outperformed the Amazonian biome, where only 540 species were found. However, in terms of their species composition, the inselbergs of both the equatorial humid Amazonian and the tropical humid Atlantic Forest biomes differ from those of the Caatinga biome in having a higher number of exclusive species (singletons) per inselberg. It has been suggested that many of the lithophytes in granite outcrops in Venezuela belong to families that contribute highly to the epiphytic Neotropical flora (e.g., Orchidaceae, Bromeliaceae, Piperaceae) (Gröger, 2000). This appears to also be true in the Atlantic Forest biome, especially when taking into account the high diversity of epiphyte-bearing groups such as Cactaceae, Bromeliaceae and Orchidaceae in the region (Menini Neto et al., 2016). Unlike the dry vegetation matrix of the Caatinga biome, where epiphytes are scarce, the Atlantic and Amazonian forests may owe part of their diversity to the exchange of epiphytic/rupicolous lineages of these plant groups.

Similar to what was found in the patchy, more seasonal Amazonian savannas (Devecchi et al., 2020), the Amazonian inselbergs also have low similarity when compared to each other, forming a less cohesive group. This may be due partly to the small size of our sample when considering the sheer size of the biome. The relatively close relationship between the two Brazilian Amazon inselbergs (PA-PHR and MT-PC) may be explained by their location at the edge of the central plateau of Brazil, while all the other Amazonian groups are located at the limit of the Guayana Shield (SUR-SIP, SUR-SR, and SUR-VOL and GF-FG, GF-MCH, GF-NOU, and GF-TRI). It is important to note that these two groups are separated by the Amazon basin lowland. It is still uncertain whether Amazonian rivers influence the flora of their inselbergs, given that Amazonian rivers have been suggested as the main drivers of current biogeographic patterns in the Amazonian biome (Patton et al., 1994; Hall and Harvey, 2002; Nazareno et al., 2017). We know that rivers do not usually serve much as barriers to inselbergs, at least for Bromeliaceae in the Caatinga and Atlantic Forest biomes (Gonçalves-Oliveira et al., 2017; Hmeljevski et al., 2017, 1).

A similar pattern of biome identity was revealed by our megatree, where little overlap between lineages and clades was found for inselbergs in different biomes. Some plant families merit further discussion. *Bromeliaceae, Orchidaceae, Cactaceae* and *Piperaceae* have rather poor representation in terms of lineages in the Amazonian inselbergs and in the Caatinga sites (except *Cactaceae*), suggesting that there may be a transition between the high species richness of epiphytes of the Atlantic Forest biome (Menini Neto et al., 2016) and the nearby harsh inselberg surfaces, but this pattern is not repeated in the other two biomes for the same families. The saxicolous Bromeliaceae seem to play a more relevant role in Atlantic Forest communities (de Paula et al., 2021). Myrtaceae and Solanaceae are better represented in the Atlantic Forest, with Myrtaceae following the trend revealed by recent taxonomic research (Lucas and Bünger, 2015).

While *Poaceae* and *Cyperaceae* were among the richest families in the Amazonian inselbergs, the lineage representation shows that certain clades of Poaceae (tribe Paniceae) were more common in the Amazonian biome, while others were more common in the Caatinga biome (subfamily *Chloridoideae*). For *Cyperaceae*, on the other hand, there was a predominance of the tribe *Cyperae* in the Caatinga, while *Schoeneae* (represented by *Rhynchospora*) was better represented in the Amazonian inselbergs. Some monocot families, such as Eriocaulaceae and Xyridaceae, which are very diverse in Eastern Brazil *campo rupestre* quartzitic and ferruginous outcrops (Zappi et al., 2017; Andrino et al., 2021), are surprisingly absent from Atlantic Forest inselbergs. This may be additional evidence of the importance of the biome in which an inselberg is located for the occurrence of certain lineages, or perhaps their relative absence is connected with their inability to establish and thrive in granitic substrates.

Noticeably less represented in the Amazon than in the other Neotropical biomes, the pattern shown by the Asteraceae in the inselbergs coincides with the general pattern observed for the biome, where only a few genera are of any notice, such as *Ichthyothere* and *Riencourtia*. However, while Asteraceae was reasonably well represented in the Caatinga inselbergs, primarily by *Vernonieae* and *Heliantheae*, it was almost absent in the Atlantic Forest inselbergs, with the clades corresponding to Mikania (*Astereae*) and Baccharis (*Eupatorieae*) being represented instead.

*Euphorbiaceae, Fabaceae (Leguminosae)* and *Malvaceae* were the most commonly represented families in the inselbergs in all three biomes. However, the lineages represented have hardly any commonality, and *Fabaceae* particularly had important clades (*Ingeae* and *Dalbergieae*) present in only the Atlantic Forest biome, while other taxa (the genus *Mimosa* and tribe Cassieae) were probably specialized for the drier conditions of the Caatinga inselbergs. Melastomataceae has a more even spread of lineages in the Amazonian inselbergs, while in the Caatinga inselbergs, their representation is limited, with several clades missing the Miconieae tribe and *Marcetia* alliance (Michelangeli et al., 2013). Among the richest families from only the Caatinga inselbergs, *Convolvulaceae* contributes to the diversity through specific lineages and clades (*Merremieae* and *Ipomoeeae*). Lamiaceae was also more common in the Caatinga inselbergs than in the other biomes.

### Precipitation as a driver of compositional turnover

Species turnover was mostly explained by predictors related to precipitation (44.81%). Precipitation patterns are known to affect vital physiological processes in flowering plants, such as germination, seedling establishment and growth, photosynthesis and biomass accumulation, potentially imposing selection on species traits and composition (Fay and Schultz, 2009; Yu et al., 2015; Martins et al., 2019). Climatic variables such as precipitation also explained much about diversity in the inselberg communities in the Atlantic Forest and in the Southwest Australia Floristic Region (Yates et al., 2019; de Paula et al., 2021). Patterns of annual rainfall are also markedly different in distinct biomes (Alvares et al., 2013), potentially reinforcing that matrix permeability is linked to variations in rainfall. Geographic distance, usually related to dispersal limitations, also explained a large proportion of species turnover (27.58%). This could signify that the lower similarity found among the Amazonian inselbergs is due to their geographical isolation when compared to sampled inselbergs within the Caatinga or Atlantic Forest biomes. Therefore, future studies assessing more inselbergs within Amazonia are needed to clarify similarity patterns and matrix permeability in this biome. Other investigations of beta diversity patterns in arid habitats within South America have highlighted water availability as one of the most relevant environmental drivers of species turnover (Neves et al., 2015; Bueno et al., 2018; Silva and Souza, 2018). Nevertheless, these investigations attributed less relevance to precipitation, and none found water availability to be the only relevant climatic predictor shaping species distribution, potentially indicating that precipitation patterns pose a greater challenge for plant establishment in inselbergs than in other arid environments, which could lead to the high permeability of arid lowlands.

### Environmental filtering across three biomes

Research developed in French Guiana inselbergs (Henneron et al., 2019) compared the influence of inselberg size, isolation, environmental (altitude, rainfall) and dispersal filtering on inselberg diversity, concluded that this was strongly influenced by inselberg size and environmental factors. Unfortunately we could not retrieve inselberg size for the present work as we used literature sources and such measurements were either not available or not comparable. Henneron et al. (2019) focused on Amazonian inselbergs and did not involve different biomes, however both studies coincide in pointing at rainfall as a diversity driver. Despite the fact that geographic distance played an important role in our work, Henneron et al. (2019) did not single out this variable as important for determining the diversity found in the inselbergs they studied. In our case, it is possible that the geographic distance is a proxy for the biomes, which we found influence the flora found in each group of inselbergs.

In terms of South American processes and patterns of diversification, the theories and hypotheses presented by Rull and Vegas-Vilarrúbia (2020) for the Pantepuis fit better with other *campos rupestres* from ancient Eastern Brazilian highlands and, to a lesser extent, the *campos de altitude*. The inselbergs studied here fall between sea level and 600 m a.s.l., and it is possible that past fluctuations in drought/drier conditions have played a role, but it is very unlikely that these underwent freezing temperatures caused total extinction of the local flora. Such fluctuations might also have created space for the colonization and recolonization of the exposed rocks, with species recruitment from nearby areas of drier forest (hence the biome identity we found here). The Distance dispersal theory (DDT) and the Specialized habitat theory (SHT) seem plausible for these lowland inselbergs (Mayr and Phelps, 1967). The Continuum multifactor hypothesis (CMH, Rull, 2011) also may explain the complex processes leading to the selection of the different floras of the inselbergs according to the biomes where they are included.

### Mostly unprotected habitats harboring potentially important drought-resistant plant species

**Figure 7:**
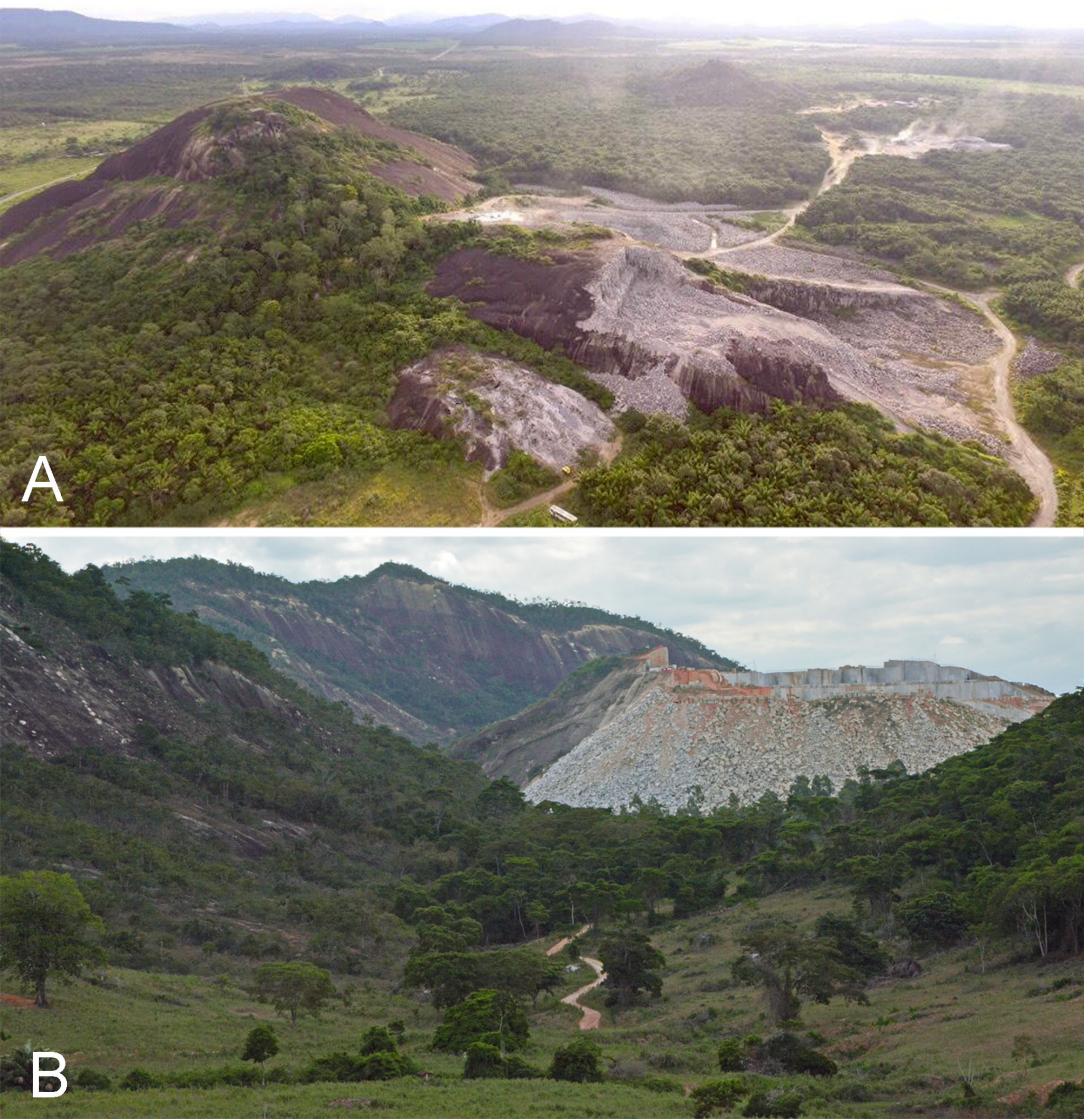
Intense threats to South America's inselbergs. **(A)** Aerial view of mining in Mucajaí municipality, Roraima state (Amazonian biome). **(B)** Mining practices threatening inselbergs on the road to Águia Branca municipality, Espírito Santo state (Atlantic Forest biome). Photos: Rafael Grisostenes **(A)**; Luísa Azevedo **(B)**.

The representation of the studied inselbergs within protected areas is uneven and raises conservation concerns. Of all 50 inselbergs included in this analysis, slightly more than 30% are found in protected areas, with a larger proportion in Amazonia, less than half in the Atlantic Forest and none in the studied sites in the Caatinga. Since there is uniqueness in each inselberg and different lineages of drought-resistant plants are found in each group of inselbergs, there is a critical need to preserve these environments to maintain the balance of this ecosystem and its surroundings. Inselbergs are often seen as commercial sources of exploitable rock and transformed into quarries (**Figure 7**) or become degraded through the destruction of the surrounding vegetation matrix due to the arrival of invasive plants (Porembski et al., 2016). In the Amazonian biome, the exploitable inselbergs are similar to those already reported for the Atlantic Rainforest (de Paula et al., 2020). Granite mines also exist near the PHR in the Carajás National Forest (ICMBio, 2016). Aditionally, the municipality of Parauapebas has plans to boost toursim in the PHR inselberg, with a private concession of the area for developments which may threaten its restricted flora. Such type of private concession has been shown to cause environmental impacts in other areas (Gomes et al., 2022) and may now pose a threat to this area.

Due to the distinct floristic and phylogenetic structure of inselbergs in different biomes, there is a very strong need to include these formations in protected areas and, in fact, create protected Caatinga inselbergs, as they are underrepresented. Historically, the Caatinga biome has been perceived as less biodiverse and less valuable than the surrounding biomes and has received less protection, having a much smaller proportion of its territory protected (Teixeira et al., 2021) when considering all the Brazilian territory. This situation is reflected by the alarming realization that none of the studied Caatinga outcrops is legally protected.

## Conclusion

This is the most comprehensive comparative study of South American inselbergs to date, and the large dataset compiled for this study reveals that, despite being similar at a first glance, the flora of these landscapes is rather distinctive in terms of floristic composition. The dissimilarity between biomes suggests the inselbergs have a different biogeographical histories unfolded even under comparable environmental filtering. The floras of the inselbergs present a congruent pattern with the biome where they are located, with low species sharing between inselbergs of different biomes. This pattern occurs even when the outcrops microclimatic conditions differ strongly from those of the surrounding matrix. The flora of the Caatinga inselbergs appears to have played an important role in linking the other biomes. It is possible that plants were able to colonize rather distant inselbergs even when these are found under very different climatic conditions, as observed for the Amazonian and Atlantic Forest biomes.

Future pathways highlighted by this study are the need to gather data and evaluate the influence of microclimatic conditions and geomorphology on the inselberg plant community structure using data loggers and unoccupied aerial vehicles (UAVs or drones). Additionally, the divergence of phylogenetic lineages found on the inselbergs in each biome stimulates future studies regarding evolutionary history and biogeography of rupicolous flora and on its functional traits through niche conservatism. As next steps, we will investigate the strong dissimilarity between inselberg flora across the three biomes by looking at species dispersal mechanisms and gene flow of plant populations. Studies of indicator and edaphic endemic species are also under way. It is paramount to investigate to what extent inselbergs can be considered islands, especially through the role of the vegetation matrix in their plant community. Futhermore, there is an urgent need to undertand the effects of global climate change on the inselberg flora, as these ecosystems may be close to the heat and drought limit due to their shallow soils and exposure to the elements. Regarding inselberg conservation, we need to take steps to set the Caatinga as a priority, as none of the inselbergs evaluated in this study falls within protected areas.

## Data availability statement

The datasets presented in this study can be found in online repositories. The names of the repository/repositories and accession number(s) can be found in the article/**Supplementary Material**.

## Author contributions

RB-S, CA, JL, AH, PV and DZ designed the study, collected and identified material from the newly sampled area and wrote the first draft of the manuscript. RB-S, CA, JL, AH, DZ and LA compiled the data. RB-S and CA performed multivariate analyses, LA and JL prepared the phylogenetic tree, and LL fitted the generalized dissimilarity model. The manuscript was written and edited by RB-S, CA, JL, LA, LL, PV, TG and DZ. All authors contributed to the article and approved the submitted version.

## Funding

CNPQ productivity and doctoral fellowships, CAPES doctoral fellowships, ITV project support.

## Acknowledgments

We thank the curators of the MG, BHCB, and HCJS herbaria. DZ and PV are holders of CNPq productivity grants (304178/2021-7 and 312486/2020-0, respectively). LA and LL acknowledge financial support from the Coordination for the Improvement of Higher Education Personnel (CAPES – finance code 001). The authors wish to thank César de Sá Carvalho Neto, Delmo Silva and Lourival Tyski from the BioParque Vale Amazônia, in Carajás for logistic support. This work is supported by the project “Natural Capital - R100603.83” of the Instituto Tecnológico Vale and Museu Paraense Emílio Goeldi.

## Conflict of interest

The authors declare that the research was conducted in the absence of any commercial or financial relationships that could be construed as a potential conflict of interest.

## Publisher’s note

All claims expressed in this article are solely those of the authors and do not necessarily represent those of their affiliated organizations, or those of the publisher, the editors and the reviewers. Any product that may be evaluated in this article, or claim that may be made by its manufacturer, is not guaranteed or endorsed by the publisher.
