# Supplementary material for: A wide range of South American inselberg floras reveal cohesive biome patterns
Source: Front Plant Sci. 2022 Sep 29;13:928577. doi: 10.3389/fpls.2022.928577 (PMC9559578; doi:10.3389/fpls.2022.928577)
Supplement: Supplementary file 3 [file Table_3.docx]

**Supplementary Information**

Table 2.Vascular plant species from Pedra da Harpia.

| **Taxa** | **Life form** | **Voucher** | **Herbarium** |
| --- | --- | --- | --- |
| **Acanthaceae** |  |  |  |
| *Justicia birae* A.S. Reis, F.A. Silva, A. Gil & Kameyama | Herb | DCZ 4634 | MG |
| **Alstroemeriaceae** |  |  |  |
| *Bomarea edulis* (Tussac) Herb. | Herb | RGBS 1419 | MG |
| **Annonaceae** |  |  |  |
| *Annona exsucca* DC. | Tree | RGBS 1276 | MG |
| **Apocynaceae** |  |  |  |
| *Aspidosperma multiflorum* A. DC. | Treelet | RGBS 1357 | MG |
| *Mandevilla scabra* (Hoffmanns. ex Roem. & Schult.) K.Schum. | Liana | AJA 332 | BHCB |
| *Marsdenia bergii* Morillo | Liana | RGBS 1344 | MG |
| **Araceae** |  |  |  |
| *Philodendron acutatum* Schott | Herb | DCZ 4665 | MG |
| *Scaphispatha robusta* E.G. Gonç. | Herb | RGBS 1355 | MG |
| **Asteraceae** |  |  |  |
| *Bidens pilosa* L. | Herb | AJA 216 |  |
| *Ichthyothere* sp. | Shrub |  |  |
| *Lepidaploa remotiflora* (Rich.) H. Rob. | Shrub | DCZ 4650 | MG |
| *Riencourtia latifolia* Gardner | Herb | RGBS 1412 | MG |
| *Riencourtia pedunculosa* (Rich.) Pruski | Herb | DCZ 4640 | MG |
| **Begoniaceae** |  |  |  |
| *Begonia saxicola* A.DC. | Herb | DCZ 4648 | MG |
| **Bignoniaceae** |  |  |  |
| *Adenocalymma schomburgki*i (DC.) L.G.Lohmann | Liana | RGBS 1275 | MG |
| *Fridericia japurensis* (DC.) L.G. Lohmann | Liana | DCZ 4639 | MG |
| **Bixaceae** |  |  |  |
| *Cochlospermum regium* (Schrank) Pilg. | Tree | TLFS 115 |  |
| **Bromeliaceae** |  |  |  |
| *Ananas ananassoide*s (Baker) L.B.Sm. | Herb | DCZ 4666 | MG |
| *Pitcairnia burchelli*i Mez | Herb | RGBS 13 41 | MG |
| **Cactaceae** |  |  |  |
| *Cereus hexagonus* (L.) Mill. | Herb | DCZ 3534 | MG |
| **Caryophyllaceae** |  |  |  |
| *Drymaria cordata* (L.) Willd. ex Roem & Schult. | Herb | DCZ 3848 | MG |
| **Celastraceae** |  |  |  |
| *Anthodon decussatus* Ruiz & Pav. | Liana | DCZ 4676 | MG |
| **Chrysobalanaceae** |  |  |  |
| *Hirtella lancifolia Ducke* | Tree | RGBS 1278 | MG |
| **Combretaceae** |  |  |  |
| *Combretum laxum* Jacq. | Liana | DCZ 4638 | MG |
| **Convolvulaceae** |  |  |  |
| *Distimake macrocalyx* (Ruiz & Pav.) A.R. Simões & Staples | Liana | TLFS 116 |  |
| *Evolvulus filipes* Mart. | Liana | DCZ 4652 | MG |
| *Jacquemontia tamnifolia* (L.) Griseb. | Liana | DCZ 4651 | MG |
| *Operculina hamiltonii* (G.Don) D.F.Austin & Staples | Liana | DCZ 4642 | MG |
| **Costaceae** |  |  |  |
| *Chamaecostus lanceolatus* (Petersen) C.D.Specht & D.W.Stev. | Herb | RGBS 1353 | MG |
| **Cucurbitaceae** |  |  |  |
| *Siolmatra brasiliensis* (Cogn.) Baill. | Liana | RGBS 1343 | MG |
| **Cyperaceae** |  |  |  |
| *Cyperus* sp. | Herb | LMMC 1150 | MG |
| *Rhynchospora pubera* (Vahl) Boeckeler | Herb | AJA 775 | BHCB |
| *Rhynchospora seccoi* C.S. Nunes, P.J.S. Silva Filho & A. Gil | Herb | CFH 1144 | MG |
| *Scleria verticillata* Muhl. ex Willd. | Herb | CFH 1150 | MG |
| **Dichapetalaceae** |  |  |  |
| *Dichapetalum rugosum* (Vahl) Prance | Shrub/Treelet | DCZ 4632 | MG |
| **Dioscoreaceae** |  |  |  |
| *Dioscorea asperula* Pedralli | Liana | RGBS 1420 | MG |
| *Dioscorea campestris* Griseb. | Liana | RGBS 1342 | MG |
| *Dioscorea piperifolia* Humb. & Bonpl. ex Willd. | Liana | RGBS 1416 | MG |
| *Dioscorea planistipulosa* Uline ex R.Knuth | Liana | RGBS 1340 | MG |
| *Dioscorea subhastata* Vell. | Liana | RGBS 1417 | MG |
| **Erythroxylaceae** |  |  |  |
| *Erythroxylum rufum* Cav. | Shrub/Treelet | RGBS 1346 | MG |
| **Euphorbiaceae** |  |  |  |
| *Croton cajucara* Benth | Shrub | AJA 214 |  |
| *Dalechampia peckoltiana* Mull.Arg | Liana | DCZ 4663 | MG |
| *Dalechampia tiliifolia* Lam. | Liana | AJA 328 |  |
| *Microstachys salicifolia* (Mart.) [Pscheidt & Cordeiro ex] M.J.Silva | Shrub | PBM 1150 |  |
| *Mabea angustifolia* Spruce ex Benth. | Treelet | LGS 2 | MG |
| *Manihot quinquepartita* Huber ex D.J.Rogers | Shrub | RGBS 1347 | MG |
| *Manihot tristis* Müll.Arg. | Shrub | DCZ 4639 | MG |
| *Sapium argutum* (Müll.Arg.) Huber | Shrub | RGBS 1354 | MG |
| **Gesneriaceae** |  |  |  |
| *Drymonia serrulata* (Jacq.) Mart. | Shrub | RGBS 1423 | MG |
| **Heliconiaceae** |  |  |  |
| *Heliconia psittacorum* L.f. | Herb | RGBS 1406 | MG |
| **Hypericaceae** |  |  |  |
| *Vismia tenuinervia* (M.E.Berg) N.Robson | Tree | RGBS 1345 | MG |
| **Iridaceae** |  |  |  |
| *Alophia graniticola* A. Gil | Herb | AG 601 | MG |
| **Lamiaceae** |  |  |  |
| *Mesosphaerum suaveolens* (L.) Kuntze | Herb | AJA 777 | MG |
| **Leguminosae** |  |  |  |
| *Anadenanthera peregrina* (L.) Speg. | Tree | HCL 7048 |  |
| *Bauhinia piresii* Vaz & G.P. Lewis | Liana | RGBS 1414 | MG |
| *Bauhinia pulchella* Benth. | Shrub | DCZ 4900 | MG |
| *Centrosema grazielae* V.P.Barbosa | Liana | DCZ 4656 | MG |
| *Copaifera martii* Hayne | Tree | DCZ 4671 | MG |
| *Dalbergia spruceana* Kunth | Tree | DCZ 4669 | MG |
| *Dioclea apurensis* Kunth | Liana | RGBS 1418 | MG |
| *Dioclea guianensis* Benth. | Liana | DCZ 4896 | MG |
| *Mimosa somnians* Humb. & Bonpl. ex Willd. | Shrub | DCZ 4898 | MG |
| *Senegalia multipinnata* (Ducke) Seigler & Ebinger | Tree | DCZ 4660 | MG |
| *Senegalia polyphylla* (DC.) Britton & Rose | Tree | DCZ 4893 | MG |
| *Swartzia arumateuana* (R. S. Cowan) Torke & Mansano | Treelet | DCZ 4894 | MG |
| **Malpighiaceae** |  |  |  |
| *Banisteriopsis malifolia* (Nees & Mart.) B.Gates | Liana | DCZ 4662 | MG |
| *Banisteriopsis stellaris* (Griseb.) B.Gates | Liana | DCZ 4678 | MG |
| *Heteropterys dumetorum* (Griseb.) Nied. | Liana | RGBS 1348 |  |
| *Peixotoa reticulata* Griseb. | Liana | DCZ 4654 |  |
| **Malvaceae** |  |  |  |
| *Helicteres brevispira* A.St.-Hil. | Shrub | RGBS 1349 | MG |
| *Helicteres eitenii* Leane | Shrub | RGBS 1351 | MG |
| *Luehea candicans* Mart. & Zucc. | Tree | RGBS 1352 | MG |
| *Pachira tocantina* (Ducke) Fern. Alonso | Shrub | RGBS 1274 | MG |
| *Pavonia malacophylla* (Link & Otto) | Shrub | DCZ 4895 | MG |
| *Pseudobombax longiflorum* (Mart.) A.Robyns | Treelet | RMH 57925 | MG |
| *Sida linifolia* Cav. | Subshrub | DCZ 4653 | MG |
| **Marantaceae** |  |  |  |
| *Goeppertia* sp. | Herb | DCZ 4675 | MG |
| *Maranta friedrichsthaliana* Körn. | Herb | RGBS 1407 | MG |
| **Melastomataceae** |  |  |  |
| *Brasilianthus carajensi*s Almeda & Michelangeli | Herb | RGBS 1422 | MG |
| *Miconia heliotropoides* Triana | Shrub | AAO 4540 |  |
| *Miconia nervosa* (Sm.) Triana | Shrub | AAO 4529 |  |
| *Pseudoernestia cordifolia* O.Berg ex Triana | Shrub | RGBS 1408 | MG |
| *Pterolepis trichotoma* (Rottb.) Cogn. | Shrub | DCZ 4681 | MG |
| *Tibouchina* sp. nov. | Shrub/Treelet | DCZ 4645 | MG |
| **Myrtaceae** |  |  |  |
| *Eugenia citrifolia* Poir. | Shrub | RGBS 1350 | MG |
| **Opiliaceae** |  |  |  |
| *Agonandra silvatica* Ducke | Treelet | RGBS 1277 | MG |
| **Orchidaceae** |  |  |  |
| *Cyrtopodium andersonii* (Lamb. ex Andrews) R.Br. | Herb | RMH 57924 | MG |
| **Passifloraceae** |  |  |  |
| *Passiflora coccinea* Aubl. | Liana | AAJ 320 |  |
| **Phyllanthaceae** |  |  |  |
| *Phyllanthus minutulus* Müll.Arg. | Herb | DCZ 4643 | MG |
| **Poaceae** |  |  |  |
| *Hildaea tenuis*  (J. Presl & C.Presl) C.Silva & R.P.Oliveira | Herb | DCZ 4679 | MG |
| *Ichnanthus calvescens* (Nees ex Trin.) Döll | Herb | AJA 336 |  |
| *Lasiacis ligulata* Hitchc. & Chase | Herb | DCZ 4901 | MG |
| *Rugoloa pilosa* (Sw.) Zuloaga | Herb | RGBS 1413 | MG |
| *Sporobolus temomairemensis* Judz. & P.M.Peterson | Herb | PLV 5270 |  |
| *Trichanthecium arctum* (Swallen) Zuloaga & Morrone | Herb | AJA 334 |  |
| **Polygalaceae** |  |  |  |
| *Caamembeca spectabilis* (DC.) J.F.B.Pastore | Shrub | RGBS 1356 | MG |
| *Polygala paniculata* L. | Herb | DCZ 4664 | MG |
| **Primulaceae** |  |  |  |
| *Clavija macrophylla* (Link ex Roem. & Schult.) Miq. | Shrub | DCZ 4633 | MG |
| **Rhamnaceae** |  |  |  |
| *Gouania frangulifolia* (Willd. ex Roem. & Schult.) Radlk. | Liana | AJA 215 |  |
| **Rubiaceae** |  |  |  |
| *Borreria heteranthera* E.L.Cabral & Sobrado | Herb | DCZ 4646 | MG |
| *Borreria ocymifolia* (Roem. & Schult.) Bacigalupo & E.L.Cabral | Herb | AJA 319 |  |
| *Borreria paraensis* E.L.Cabral & Bacigalupo | Herb | LFAP 465 |  |
| *Coutarea hexandra* (Jacq.) K.Schum. | Tree | DCZ 4672 | MG |
| *Mitracarpus carajasensis E.L. Cabral, Sobrado & E.B. Souza* | Herb | PLV 5277 |  |
| *Palicourea guianensis* Aubl. | Shrub | LFAP 467 |  |
| *Spermacoce* sp. | Herb | AJA 778 | BHCB |
| **Rutaceae** |  |  |  |
| *Esenbeckia pilocarpoides* Kunth | Shrub/Treelet | DCZ 4636 | MG |
| *Pilocarpus carajaensis* Skorupa | Shrub | DCZ 4635 | MG |
| **Sapindaceae** |  |  |  |
| *Serjania lethalis* A.St.-Hil. | Liana | DCZ 4680 | MG |
| **Solanaceae** |  |  |  |
| *Solanum asperum* Rich. | Shrub | AJA 317 | BHCB |
| *Solanum crinitum* Lam. | Shrub | AJA 217 | BHCB |
| *Solanum rugosum* Dunal | Shrub | DCZ 4658 | MG |
| **Turneraceae** |  |  |  |
| *Turnera laciniata* Arbo. | Shrub | DCZ 4657 | MG |
| **Urticaceae** |  |  |  |
| *Cecropia palmata* Willd. | Tree | DCZ 4899 | MG |
| **Velloziaceae** |  |  |  |
| *Vellozia glauca* Pohl | Herb | RGBS 1405 | MG |
| **Verbenaceae** |  |  |  |
| *Lantana cujabensis* Schauer | Shrub | AJA 330 | BHCB |
| **Vitaceae** |  |  |  |
| *Cissus apendiculata* Lombardi | Liana | DCZ 4661 | MG |
| *Cissus tinctoria* Mart. | Liana | RGBS 1415 | MG |
