# Supplementary material for: A wide range of South American inselberg floras reveal cohesive biome patterns
Source: Front Plant Sci. 2022 Sep 29;13:928577. doi: 10.3389/fpls.2022.928577 (PMC9559578; doi:10.3389/fpls.2022.928577)
Supplement: Supplementary file 5 [file Table_5.docx]

########################################

#Barbosa-Silva et al. 2022 #

# #

#A wide range of South American #

#inselberg floras reveals cohesive #

#biome patterns #

# #

#Frontiers in Plant Science #

# #

########################################

###########################################################################################################

### I. Similarity patterns among investigated areas

library(vegan)

# 1. Import species dataset (areas + biome + binary matrix w/ presence x absence)

sim.esp = read.csv(file="areas x spp.csv",header=TRUE,sep=";", row.names = 1)

View(sim.esp)

# 1.1. Segment dataset (binary matrix and biomes)

class <- sim.esp[,1]

spp <- sim.esp[,2:2197]

spp[,][spp[,] >0] <- 1 # in case the presence x absence matrix is not binary

# 2. Measure dissimilarity among studied areas

dist.bray <- vegdist(spp, method="bray") # using Sorensen as the dissimilarity measure

dist.bray

# 3. NMDS

NMDS<- metaMDS(dist.bray, distance="bray", k=2, trymax=50000)

NMDS

plot(NMDS, display="sites",cex=.9, type="t")

# 4. UPGMA

hclust(dist.bray, method="average")

UPGMA<- hclust(dist.bray, method="average")

UPGMA

plot(UPGMA,hang=-1,cex=0.9, col="black")

# 5. ANOSIM

groupsim <- anosim(spp, class, permutations= 999, distance= "bray")

groupsim

plot(groupsim, main= "ANOSIM")

###########################################################################################################

### II. Compile (and export) shared species datasets

## A) AMONG BIOMES

# 1. Import binary matrix with species presence x absence in each biome

m = read.csv(file="biomes x spp.csv", header=TRUE, sep=";", row.names = 1)

m[m > 0] <- 1

# 2. Compile number of shared species (absolute)

sim <- matrix(ncol = 3, nrow = 3)

vec <- numeric(1)

for (j in 1:3) {

for (i in 1:3) {

vec[i] <- sum(m[j,]>0 & m[i,]>0)

}

sim[j,] <- c(vec)

}

mat <- as.matrix(sim)

rownames(mat) <- rownames(m)

colnames(mat) <- rownames(m)

mat

## B) AMONG INSELBERGS

# 1. Import binary matrix with species presence x absence in each biome

sim.esp = read.csv(file="areas x spp.csv",header=TRUE,sep=";", row.names = 1)

datax <- sim.esp[,2:2197]

datax[is.na(datax)] <- 0

datax[datax > 0] <- 1

# 2. Compile number of shared species (absolute)

sim <- matrix(ncol = 50, nrow = 50)

vec <- numeric(1)

for (j in 1:50) {

for (i in 1:50) {

vec[i] <- sum(datax[j,]>0 & datax[i,]>0)

}

sim[j,] <- c(vec)

}

mat <- as.matrix(sim)

rownames(mat) <- rownames(datax)

colnames(mat) <- rownames(datax)

write.csv(mat,"sharedspp_absol.csv", row.names = TRUE) # export

# 3. Compile number of shared species (proportion)

sim <- matrix(ncol = 50, nrow = 50)

sum <- numeric(1)

vec <- numeric(1)

for (j in 1:50) {

sum[j] <- sum(datax[j,]>0)

for (i in 1:50) {

vec[i] <- ((sum(datax[j,]>0 & datax[i,]>0)*100)/sum[j])

}

sim[j,] <- c(vec)

}

prop <- matrix(ncol = 51, nrow = 51)

prop[2:51,2:51] <- sim

prop <- as.matrix(sim)

rownames(prop) <- rownames(datax)

colnames(prop) <- rownames(datax)

prop1 <- round(prop, digits=1)

write.csv(prop1,"sharedspp_prop.csv", row.names = TRUE) # export

# 4. Compile number of exclusive species (absolute)

library(dplyr)

data <- t(datax)

data <- as.data.frame(data)

data_cleaned = data[rowSums(data)!=1,]

single <- anti_join(data,data_cleaned)

rowSums(data)

rowSums(data_cleaned)

rowSums (single)

single <- t(single)

single <- as.data.frame(single)

singletons <- rowSums(single)

singletons

write.csv(singletons,"exclusivespp_abs.csv", row.names = TRUE) # export

# 5. Compile vector with the total number of species found in each inselberg

sum <- numeric(50)

for (j in 1:50) {

sum[j] <- sum(datax[j,]>0)

}

sum <- as.data.frame(sum)

rownames(sum) <- rownames(datax)

write.csv(sum,"totalspp.csv", row.names = TRUE) # export

# 6. Compile number of exclusive species (proportion)

prop_single <- singletons*100/sum

write.csv(singletons,"exclusivespp_prop.csv", row.names = TRUE) # export

data = read.csv(file="spp_areas total.csv",header=TRUE,sep=";", row.names=1)

data <- t(datax)

data <- as.data.frame(data)

data_cleaned = data[rowSums(data)!=1,]

data_cleaned <- t(data_cleaned)

data_cleaned <- as.data.frame(data_cleaned)

###########################################################################################################

### III. Heatmap

library(vegan)

library(ComplexHeatmap)

# 1. Import species dataset (areas + biome + binary matrix w/ presence x absence)

sim.esp = read.csv(file="areas x spp.csv", header=TRUE, sep=";", row.names = 1)

sim.esp1 = read.csv(file="areas x spp.csv", header=TRUE, sep=";")

# 1.1. Segment dataset (binary matrix and biomes)

area <- sim.esp1[,1:2]

spp <- sim.esp[,2:2196]

spp[,][spp[,] >0] <- 1 # in case the presence x absence matrix is not binary

# 2. Compile dendrogram (UPGMA)

dist.bray <- vegdist(spp, method="bray") # using Sorensen as the dissimilarity measure

dist.bray

hclust(dist.bray, method="average")

UPGMA <- hclust(dist.bray, method="average")

UPGMA

plot(UPGMA,hang=-1,cex=0.9, col="black")

group <- as.dendrogram(UPGMA)

# 3. Import matrixes (compiled in section II)

# 4. Construct heatmap

library(ComplexHeatmap)

library(circlize)

library(dendextend)

library(gridtext)

# 4.1. Set graphic parameters

col_fun = colorRamp2(c(0, 50, 99, 100), c("#fef0d9", "#d7301f", "#6f0000", "black")) # colors

row_dend = as.dendrogram(hclust(dist(mat)))

row_dend = color_branches(row_dend, k = 2) # dendrogram

# 4.2. Compile annotations

ha_bioma = rowAnnotation(Ecoregions = area$biome,

col = list(Ecoregions = c("Caatinga" = "#d95f02", "Amazon" = "#1b9e77", "Atlantic forest" = "#7570b3")),

gp = gpar(col = "white"),

simple_anno_size = unit(0.2, "cm"),

annotation_label = gt_render("Domain",

gp = gpar(col = "white", fontsize = 7, fontface = "bold")),

show_legend = TRUE)

ha2_bioma = HeatmapAnnotation(Ecoregions = area$biome,

col = list(Ecoregions = c("Caatinga" = "#d95f02", "Amazon" = "#1b9e77", "Atlantic forest" = "#7570b3")),

gp = gpar(col = "white"),

simple_anno_size = unit(0.2, "cm"),

annotation_label = gt_render("Domain",

gp = gpar(col = "white", fontsize = 7, fontface = "bold")),

show_legend = FALSE)

ha_area = rowAnnotation(floras = anno_text(rownames(spp), just = "left", gp = gpar(fontsize = 7)))

ha2_area = HeatmapAnnotation(floras = anno_text(rownames(spp), just = "right", gp = gpar(fontsize = 7)))

ha_exspp = rowAnnotation(ex_spp = prop_single[,1],

simple_anno_size = unit(0.28, "cm"),

col = list(ex_spp = col_fun),

annotation_label = gt_render("Exclusive spp.",

gp = gpar(col= "black", fontsize = 7, fontface = "bold", labels_rot = 45)),

gp = gpar(col = "white"),

show_legend = FALSE)

ha_n = rowAnnotation(total = anno_text(sum, gp = gpar(fontsize = 7)))

total_spp <- character(1)

for (j in 1:50) {

total_spp[j] <- paste(c("n=", sum[j,], "spp."), collapse = " ")

}

ha_totalspp = rowAnnotation(total = anno_text(total_spp, gp = gpar(fontsize = 7)))

# 4.3. Plot (and export) complete heatmap

tiff("inselberg heatmap.tiff", units="in", width=10, height=9, res=500)

Heatmap(prop1, name = "prop", col = col_fun,

rect_gp = gpar(col = "white", lwd = 0.5),

width = unit(14, "cm"), height = unit(14, "cm"),

row_names_gp = gpar(col = "white", fontsize = 8), column_names_gp = gpar(col = "black", fontsize = 8),

column_title = "column title", row_title = "Inselbergs",

column_title_gp = gpar(col = "white", fontsize = 1, fontface = "bold"),

row_title_gp = gpar(col = "black", fontsize = 10, fontface = "bold"),

cluster_columns = group, cluster_rows = group,

row_dend_reorder = FALSE, column_dend_reorder = FALSE,

column_split = 3, row_split = 3,

column_dend_side = "top", row_dend_side = "left",

left_annotation = c(ha_bioma, ha_area, ha_exspp),

right_annotation = ha_totalspp,

top_annotation = ha2_bioma,

heatmap_legend_param = list(

title = "Proportion of spp.", title_gp = gpar(fontsize = 10, fontface = "bold"))

)

dev.off()

###########################################################################################################

### IV. Chord diagram

library(circlize)

library(migest)

## A) Chord diagram of biomes

# 1. Create adjacency matrix

# 1.1. W/ observed data

df = data.frame(from = c("Amazon", "Caatinga", "Atlantic Forest", "Amazon", "Amazon", "Atlantic Forest"),

to = c("Amazon", "Caatinga","Atlantic Forest", "Atlantic Forest", "Caatinga", "Caatinga"),

value = c(540, 787, 1123, 49, 80, 149))

df

# 1.2. W/ estimated data (Chao2-shared)

df = data.frame(from = c("Amazon", "Caatinga", "Atlantic Forest", "Amazon", "Amazon", "Atlantic Forest"),

to = c("Amazon", "Caatinga","Atlantic Forest", "Atlantic Forest", "Caatinga", "Caatinga"),

value = c(1350, 1189, 2496, 179, 234, 330))

df

# 2. Indicate colors used in the links

grid.col = c("Amazon" = "#1b9e77",

"Atlantic Forest" = "#7570b3",

"Caatinga" = "#d95f02")

# 3. Plot (and export) Chord diagram

tiff("chord_biomes_estimated.tiff", units="in", width=4.8, height=4.8, res=500, bg="transparent")

circos.par (track.height=1, gap.after=5, track.margin= c(0.01,0.01))

chord <- chordDiagram(df, transparency = 0.3,

grid.col = grid.col,

annotationTrack = c("name", "grid"),

scale=TRUE,

symmetric = FALSE)

circos.clear()

dev.off()

## B) Chord diagram of inselbergs /fragmented by biome

# 1. Generate input matrix (organize areas in input table to reorder areas in the diagram)

library(dplyr)

# 1.1. Amazon

sim.esp = read.csv(file="areas x spp.csv",header=TRUE,sep=";", row.names = 1)

spp <- filter(sim.esp, biome == "Amazon")

spp <- spp[,2:2197]

sim <- matrix(ncol = 11, nrow = 11)

vec <- numeric(1)

for (j in 1:11) {

for (i in 1:11) {

vec[i] <- sum(spp[j,]>0 & spp[i,]>0)

}

sim[j,] <- c(vec)

}

df.am <- as.matrix(sim)

rownames(df.am) <- rownames(spp)

colnames(df.am) <- rownames(spp)

df.am

# 1.2. Atlantic forest

sim.esp = read.csv(file="areas x spp.csv",header=TRUE,sep=";", row.names = 1)

spp <- filter(sim.esp, biome == "Atlantic forest")

spp <- spp[,2:2197]

sim <- matrix(ncol = 14, nrow = 14)

vec <- numeric(1)

for (j in 1:14) {

for (i in 1:14) {

vec[i] <- sum(spp[j,]>0 & spp[i,]>0)

}

sim[j,] <- c(vec)

}

df.af <- as.matrix(sim)

rownames(df.af) <- rownames(spp)

colnames(df.af) <- rownames(spp)

df.af

# 1.3. Caatinga

sim.esp = read.csv(file="areas x spp.csv",header=TRUE,sep=";", row.names = 1)

spp <- filter(sim.esp, biome == "Caatinga")

spp <- spp[,2:2197]

sim <- matrix(ncol = 25, nrow = 25)

vec <- numeric(1)

for (j in 1:25) {

for (i in 1:25) {

vec[i] <- sum(spp[j,]>0 & spp[i,]>0)

}

sim[j,] <- c(vec)

}

df.ca <- as.matrix(sim)

rownames(df.ca) <- rownames(spp)

colnames(df.ca) <- rownames(spp)

df.ca

# 2. Indicate colors used in the links

# 2.1. Amazon

grid.col.am = c("VEN-PTO" = "#2b83ba", "CO-CT" = "#2b83ba",

"SUR-VOL" = "#56a9a4", "GF-TRI"= "#56a9a4",

"GF-FG" = "#82cf8e",

"GF-MCH" = "#6cbc99", "GF-NOU"= "#6cbc99",

"SUR-SIP" = "#98df85", "SUR-SR" = "#98df85",

"PA-HRP"="#f89759",

"MT-PC"="#d7191c")

# 2.2. Atlantic Forest

grid.col.af = c("MG-VM" = "#2b83ba",

"ES-PE" = "#6cbc99",

"ES-AM" = "#afe682",

"ES-MG" = "#c6ee7f",

"ES-CPA" = "#fff775",

"ES-PP" = "#ffe670",

"RJ-MI" = "#fec467",

"RJ-AM" = "#d7191c", "RJ-CH" = "#d7191c", "RJ-IC" = "#d7191c", "RJ-IM" = "#d7191c",

"RJ-MC" = "#d7191c", "RJ-PA" = "#d7191c", "RJ-PR" = "#d7191c")

# 2.3. Caatinga

grid.col.ca = c("BA-MA" = "#d7191c", "BA-MT" = "#d7191c",

"BA-FS" = "#df382b",

"PE-PF"= "#f4fc79",

"PE-LU" = "#ddf57c", "PE-PTR" = "#ddf57c", "PE-PC"= "#ddf57c", "PE-PG"= "#ddf57c", "PE-PAB" = "#ddf57c",

"PB-PS"= "#98df85", "PB-EB"= "#98df85", "PB-MC"= "#98df85", "PB-PXI"= "#98df85", "PB-PX"= "#98df85", "PB-PP"= "#98df85", "PB-LP"= "#98df85", "PB-EP"= "#98df85",

"PB-FZ"= "#82cf8e",

"CE-QUI"= "#4096af", "CE-MQ"= "#4096af",

"CE-SGA"= "#2b83ba", "CE-RV"= "#2b83ba", "CE-SP"= "#2b83ba", "CE-SP2"= "#2b83ba", "CE-LP"= "#2b83ba")

# 3. Plot (and export) Chord diagram

# 3.1. Amazon

tiff("chord_AM.tiff", units="in", width=4.8, height=4.8, res=500, bg="transparent")

circos.par (track.height=1, gap.after=5, track.margin= c(0.01,0.01))

chord <- chordDiagram(df.am, transparency = 0.3,

grid.col = grid.col.am,

annotationTrack = c("name", "grid"),

scale=TRUE,

symmetric = TRUE)

circos.clear()

dev.off()

# 3.2. Atlantic Forest

tiff("chord_AF.tiff", units="in", width=4.8, height=4.8, res=500, bg="transparent")

circos.par (track.height=1, gap.after=5, track.margin= c(0.01,0.01))

chord <- chordDiagram(df.af, transparency = 0.3,

grid.col = grid.col.af,

annotationTrack = c("name", "grid"),

scale=TRUE,

symmetric = TRUE)

circos.clear()

dev.off()

# 3.3. Caatinga

tiff("chord_CA.tiff", units="in", width=4.8, height=4.8, res=500, bg="transparent")

circos.par (track.height=1, gap.after=5, track.margin= c(0.01,0.01))

chord <- chordDiagram(df.ca, transparency = 0.3,

grid.col = grid.col.ca,

annotationTrack = c("name", "grid"),

scale=TRUE,

symmetric = TRUE)

circos.clear()

dev.off()

###########################################################################################################

### V. GDM

library(vegan)

library(gdm)

# 1. Import species dataset

sim.esp = read.csv(file="areas x spp.csv",header=TRUE,sep=";", row.names = 1)

spp <- sim.esp[,2:2197]

spp[is.na(spp)] <- 0

spp[spp > 0] <- 1

spp

# 2. Import climatic dataset (w/ geographical coordinates)

climdata = read.csv(file="climatic_data.csv",header=TRUE,sep=";")

rownames(climdata) <- climdata[,1]

climdata <- climdata[,-(2)]

climdata <- climdata[,-(4)]

# 3. Check VIF for included climatic variables

spp.b <- spp

spp.b[,1] <- NULL #area codes only as row names

vifmodel <- rda(spp.b ~ bio2 + bio3 + bio6 + bio15 + bio19 + bio12 + bio18, data = climdata)

vif.cca(vifmodel)

# 3.1. Remove collinear variables

climdata$bio19 <- NULL

climdata$bio3 <- NULL

climdata$bio17 <- NULL

climdata$bio13 <- NULL

climdata$bio5 <- NULL

climdata$bio14 <- NULL

climdata$bio16 <- NULL

climdata$bio9 <- NULL

climdata$bio1 <- NULL

climdata$bio11 <- NULL

# 4. Rename climatic variables

names(climdata)[names(climdata) == "bio2"] <- "Mean Diurnal Range"

names(climdata)[names(climdata) == "bio4"] <- "Temperature Seasonality"

names(climdata)[names(climdata) == "bio6"] <- "Minimum Temperature of Coldest Month"

names(climdata)[names(climdata) == "bio7"] <- "Temperature Annual Range"

names(climdata)[names(climdata) == "bio8"] <- "Mean Temperature of Wettest Quarter"

names(climdata)[names(climdata) == "bio10"] <- "Mean Temperature of Warmest Quarter"

names(climdata)[names(climdata) == "bio12"] <- "Annual Precipitation"

names(climdata)[names(climdata) == "bio15"] <- "Precipitation Seasonality"

names(climdata)[names(climdata) == "bio18"] <- "Precipitation of Warmest Quarter"

names(climdata)[names(climdata) == "elev"] <- "Elevation"

# 5. Format input data (using site-by-species format)

gdmTab <- formatsitepair(spp, bioFormat=1, dist="bray", abundance=FALSE,

sppFilter=10, siteColumn="Sampname",

XColumn= "COORX", YColumn="COORY",

predData=climdata)

# 6. Run gdm

model <- gdm(gdmTab, geo=TRUE)

str(model)

# 7. Plot species turnover x climatic variation

par(mfrow=c(1,1), mai=c(0.6, 0.6, 0.1, 0.2))

plot(model, plot.layout=c(5,3))

# 8. Render and export gdm tables

varImport <- gdm.varImp(gdmTab, geo=TRUE, fullModelOnly = FALSE, nPerm = 999,

splines = NULL, knots = NULL,

parallel = TRUE, cores = 2,

sampleSites = 1, sampleSitePairs = 1,

outFile = "varImportance_999perm")

write.table(varImport[[1]], file = "gdm_modelImport_999perm.csv") # model's significance and variance

write.table(varImport[[2]], file = "gdm_varImport_999perm.csv") # variable importance

write.table(varImport[[3]], file = "gdm_varSignif_999perm.csv") # variable significance

write.table(varImport[[4]], file = "gdm_npermut_999perm.csv") # number of permutations

###########################################################################################################

### VI. Estimate shared spp. and overall diversity in studied biomes

## A) Estimate shared species (Chao Shared)

library(SpadeR)

# 1. Import dataset of species occurrence (for datatype = incidence_raw)

sim.esp = read.csv(file="areas x spp.csv",header=TRUE,sep=";", row.names = 1)

# 2. Compile partial datasets

spp.am <- filter(sim.esp, biome == "Amazon")

spp.af <- filter(sim.esp, biome == "Atlantic forest")

spp.ca <- filter(sim.esp, biome == "Caatinga")

# 2.1. Amazon and Atlantic forest

spp.amaf <- rbind(spp.am, spp.af)

spp.amaf <- spp.amaf[,2:2197]

spp.amaf <- t(spp.amaf)

# 2.2. Amazon and Caatinga

spp.amca <- rbind(spp.am, spp.ca)

spp.amca <- spp.amca[,2:2197]

spp.amca <- t(spp.amca)

# 2.1. Atlantic forest and Caatinga

spp.afca <- rbind(spp.af, spp.ca)

spp.afca <- spp.afca[,2:2197]

spp.afca <- t(spp.afca)

# 3. Run estimator

# 3.1. Amazon and Atlantic forest

ChaoShared(spp.amaf, datatype = "incidence_raw",

units=c(11,14), se = TRUE, nboot = 200, conf = 0.95)

# 3.2. Amazon and Caatinga

ChaoShared(spp.amca, datatype = "incidence_raw",

units=c(11,25), se = TRUE, nboot = 200, conf = 0.95)

# 3.3. Atlantic forest and Caatinga

ChaoShared(spp.afca, datatype = "incidence_raw",

units=c(14,25), se = TRUE, nboot = 200, conf = 0.95)

## B) Estimate species diversity in each biome

library(SpadeR)

library(dplyr)

# 1. Import dataset of species occurrence (for datatype = incidence_raw)

sim.esp = read.csv(file="areas x spp.csv",header=TRUE,sep=";", row.names = 1)

# 1.1. Amazon

spp.am <- filter(sim.esp, biome == "Amazon")

spp.am <- spp.am[,2:2197]

spp.am[is.na(spp.am)] <- 0

spp.am[spp.am > 0] <- 1

spp.am <- t(spp.am)

# 1.2. Atlantic forest

spp.af <- filter(sim.esp, biome == "Atlantic forest")

spp.af <- spp.af[,2:2197]

spp.af[is.na(spp.af)] <- 0

spp.af[spp.af > 0] <- 1

spp.af <- t(spp.af)

# 1.3. Caatinga

spp.ca <- filter(sim.esp, biome == "Caatinga")

spp.ca <- spp.ca[,2:2197]

spp.ca[is.na(spp.ca)] <- 0

spp.ca[spp.ca > 0] <- 1

spp.ca <- t(spp.ca)

# 2. Run estimator

ChaoSpecies(spp.am, datatype = c("incidence_raw"), k = 10, conf = 0.95)

ChaoSpecies(spp.af, datatype = c("incidence_raw"), k = 10, conf = 0.95)

ChaoSpecies(spp.ca, datatype = c("incidence_raw"), k = 10, conf = 0.95)

###########################################################################################################

### VII. Geographical distance versus floristic dissimilarity

library(vegan)

library(dplyr)

# 1. Import datasets

# 1.1. Species composition (areas + biome + binary matrix w/ presence x absence)

setwd("C:/Users/luisa/Desktop/Acadêmico/Mestrado/Parte Escrita/Projetos paralelos/Paper Inselbergs/Final version")

inselb.spp = read.csv(file="areas x spp.csv",header=TRUE,sep=";", row.names = 1)

# 1.2. Geographical distance of inselbergs

setwd("C:/Users/luisa/Desktop/Acadêmico/Mestrado/Parte Escrita/Projetos paralelos/Paper Inselbergs/RDA")

climdata = read.csv(file="climatic_data.csv",header=TRUE,sep=";")

rownames(climdata) <- climdata[,1]

inselb.coord <- climdata[,2:4]

# 2. Filter datasets by biome

# 2.1. Amazon

spp.amazon <- filter(inselb.spp, biome == "Amazon")

spp.amazon <- spp.amazon[,-1]

coord.amazon <- filter(inselb.coord, Domain == "Amazon")

coord.amazon <- coord.amazon [,-1]

# 2.2. Atlantic Forest

spp.atlantic <- filter(inselb.spp, biome == "Atlantic forest")

spp.atlantic <- spp.atlantic[,-1]

coord.atlantic <- filter(inselb.coord, Domain == "Atlantic Forest")

coord.atlantic <- coord.atlantic [,-1]

# 2.3. Caatinga

spp.caatinga <- filter(inselb.spp, biome == "Caatinga")

spp.caatinga <- spp.caatinga[,-1]

coord.caatinga <- filter(inselb.coord, Domain == "Caatinga")

coord.caatinga <- coord.caatinga [,-1]

# 3. Measure geographical and floristic distances among studied areas

# 3.1. Amazon

braydist.am <- vegdist(spp.amazon, method="bray") # using Sorensen as the dissimilarity measure

braydist.am <- 1- braydist.am # convert to similarity matrix

eucldist.am <- vegdist(coord.amazon, method="euclidean") # using Euclidean as an aproximamtion of geographic distance

am <- cbind(eucldist.am, braydist.am, "#1b9e77")

# 3.2. Atlantic Forest

braydist.af <- vegdist(spp.atlantic, method="bray")

braydist.af <- 1- braydist.af

eucldist.af <- vegdist(coord.atlantic, method="euclidean")

af <- cbind(eucldist.af, braydist.af, "#7570b3")

# 3.3. Caatinga

braydist.ca <- vegdist(spp.caatinga, method="bray")

braydist.ca <- 1- braydist.ca

eucldist.ca <- vegdist(coord.caatinga, method="euclidean")

ca <- cbind(eucldist.ca, braydist.ca, "#d95f02")

# 4. Compile distances in one data frame

areas <- rbind(am,af,ca)

areas <- as.data.frame(areas)

colnames(areas) = c("geographic","floristic","color")

write.csv(areas,"geographic x floristic distances.csv", row.names = FALSE) # export

areas = read.csv(file="geographic x floristic distances.csv",header=TRUE,sep=",")

# 5. Plot the comparison of geographical and floristic distances

tiff("similarity x distance.tiff", units="in", width=6, height=6, res=500)

plot(areas$geographic, areas$floristic, col=areas$color, pch=16,

xlab= "Geographic distance", ylab= "Floristic similarity")

dev.off()

###########################################################################################################

#Phylogenetic structure

#Based on Danilo Neves' script

#https://github.com/dmneves/macroecology_course/blob/e0fe54d37a476dbed81375b0e22a51453393ae0f/phylocom

getwd()

setwd("C:/Users/LUISA/Dropbox/Inselbergs_Artigos/Meus artigos/Amazon_Inselberg_Rafa_Carol_Zappi/R/Phylocom_10.09.2022")

##in phylomatic

#execute phylocom.bat (make sure files "R20160415.new" and "ages" are in working directory)

#create a taxa.txt with the species from your database

#reconstruct the phylogenetic hypothesis

phylomatic -f R20160415.new -t taxa.txt > phy

#generate tree

phylocom bladj -f phy > tree.tre

##paste phylocom tree in R's working directory

#install and load packages

require(picante)

require(phytools)

#uploading the tree in R

tree <- read.tree('tree.tre')

is.ultrametric(tree)

is.rooted(tree)

#collapse single branches

tree.plot <- collapse.singles(tree)

#plot

#(use the programme FigTree or iTOL for better visualization)

plot(tree.plot, type = 'f', show.tip.label = F)

dev.off()

#saving the tree in a .tree file to open in FigTree or iTOL

require(ape)

write.tree(tree.plot, file = "inselberg_tree", append = FALSE,

digits = 10, tree.names = FALSE)
