# Supplementary material for: A wide range of South American inselberg floras reveal cohesive biome patterns
Source: Front Plant Sci. 2022 Sep 29;13:928577. doi: 10.3389/fpls.2022.928577 (PMC9559578; doi:10.3389/fpls.2022.928577)
Supplement: Supplementary file 6 [file Table_6.docx]

# Supplementary information

A wide range of South American inselberg flora reveals cohesive biome patterns

Rafael Gomes Barbosa-Silva^1,2^, Caroline Oliveira Andrino^1^, Luísa Azevedo^3^, Luísa Lucresia ^4^, Juliana Lovo^5^, Alice Lima Hiura^1^, Pedro Lage Viana^2^, Tereza Cristina Giannini^1,6^, Daniela Cristina Zappi^2,7*^

Corresponding author: Rafael G. Barbosa-Silva and Daniela C. Zappi

Email: [rafa.g29@gmail.com](mailto:rafa.g29@gmail.com) and danielazappi14@gmail.com

**Results**

## *Biogeographic patterns of Neotropical inselbergs*

Figure 1: Chord diagrams of each biome showing the flora shared between each inselberg analyzed. The colors represent latitudinal variations, with cooler colors representing areas toward the north and warmer colors representing areas toward the south. A. Network of Amazonian inselbergs. B. Network of Caatinga inselbergs. C. Network of Atlantic Forest inselbergs.

**
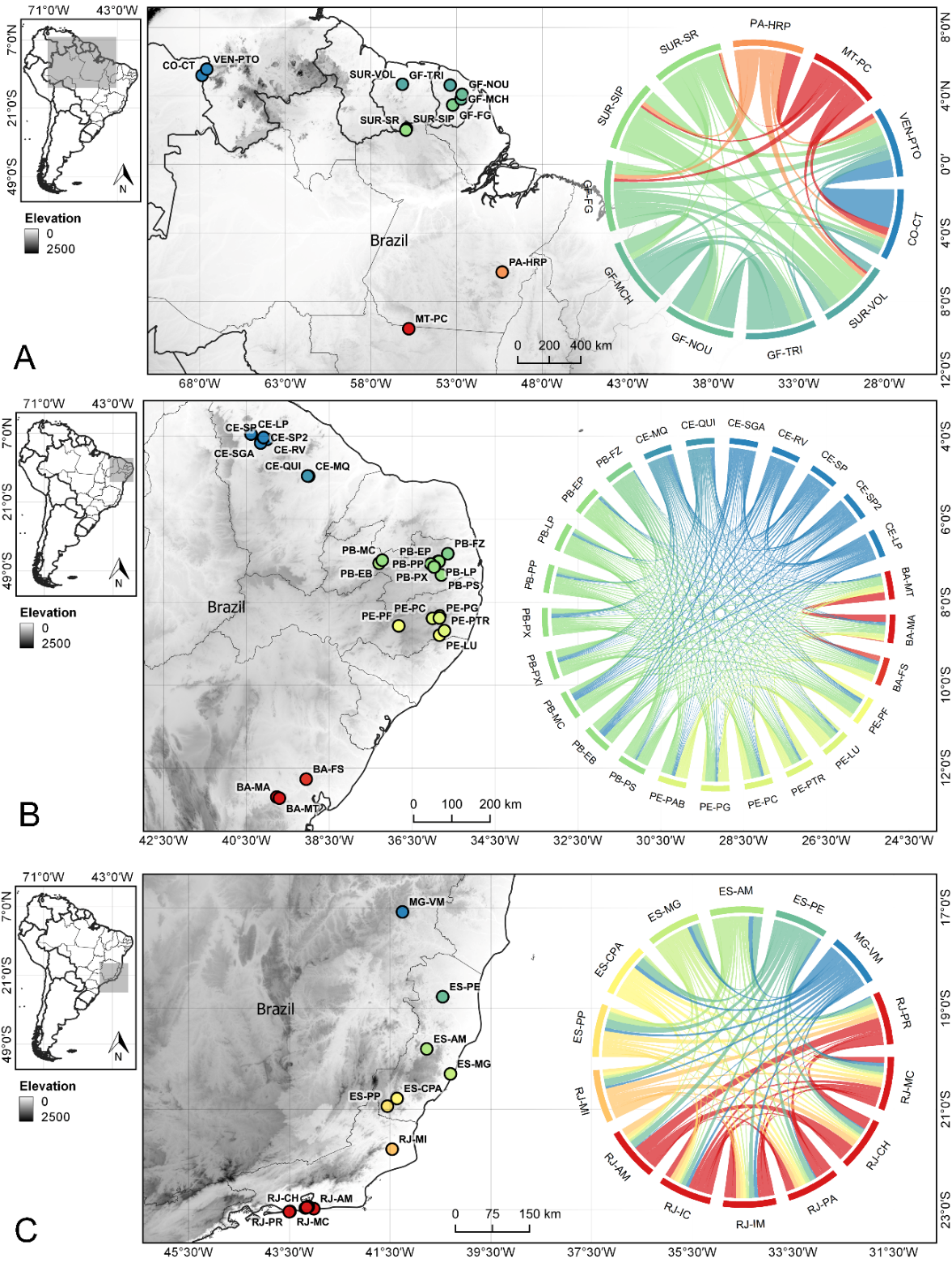
**

*Chord diagrams for estimated and observed species sharing among biomes*


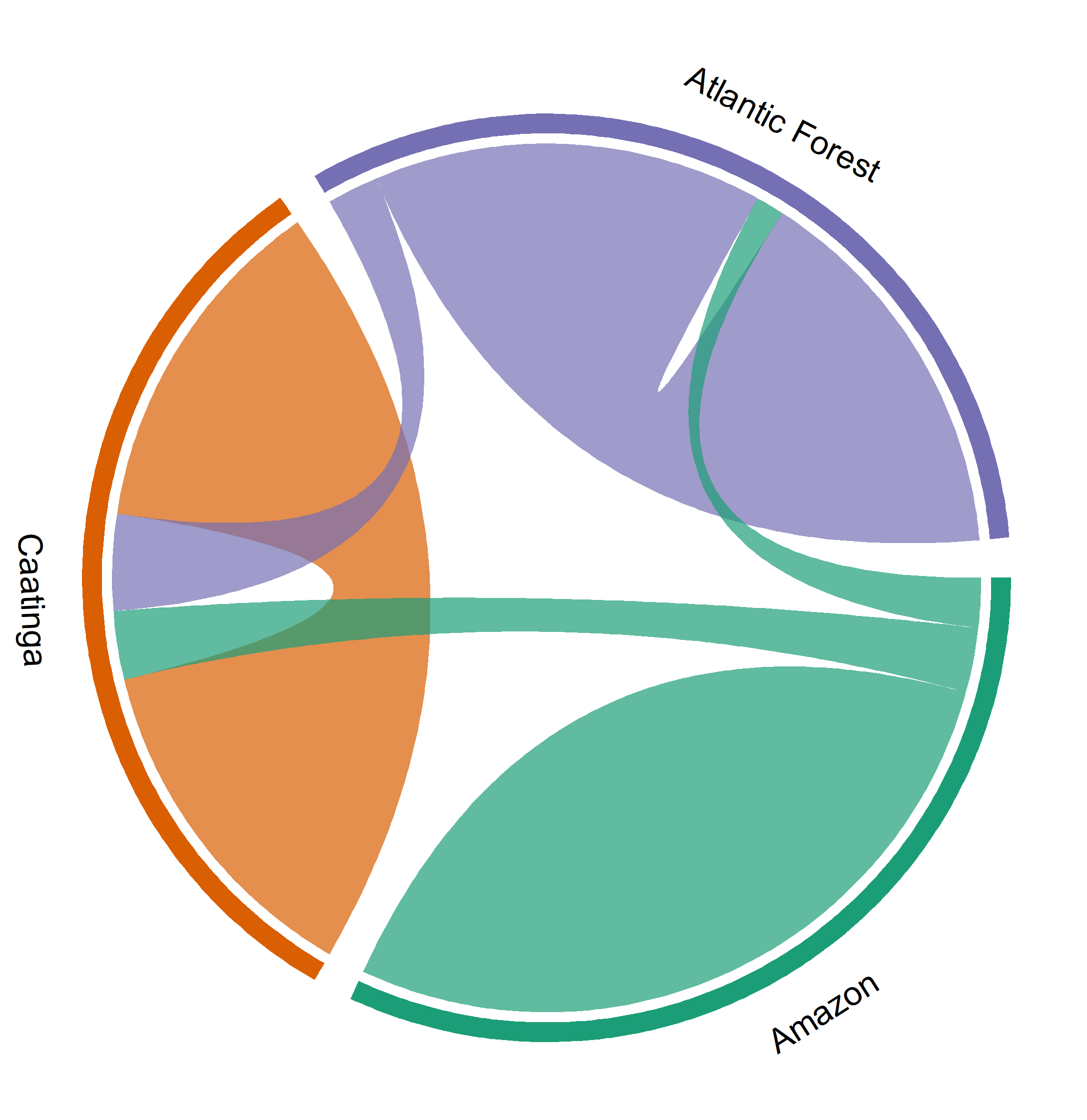

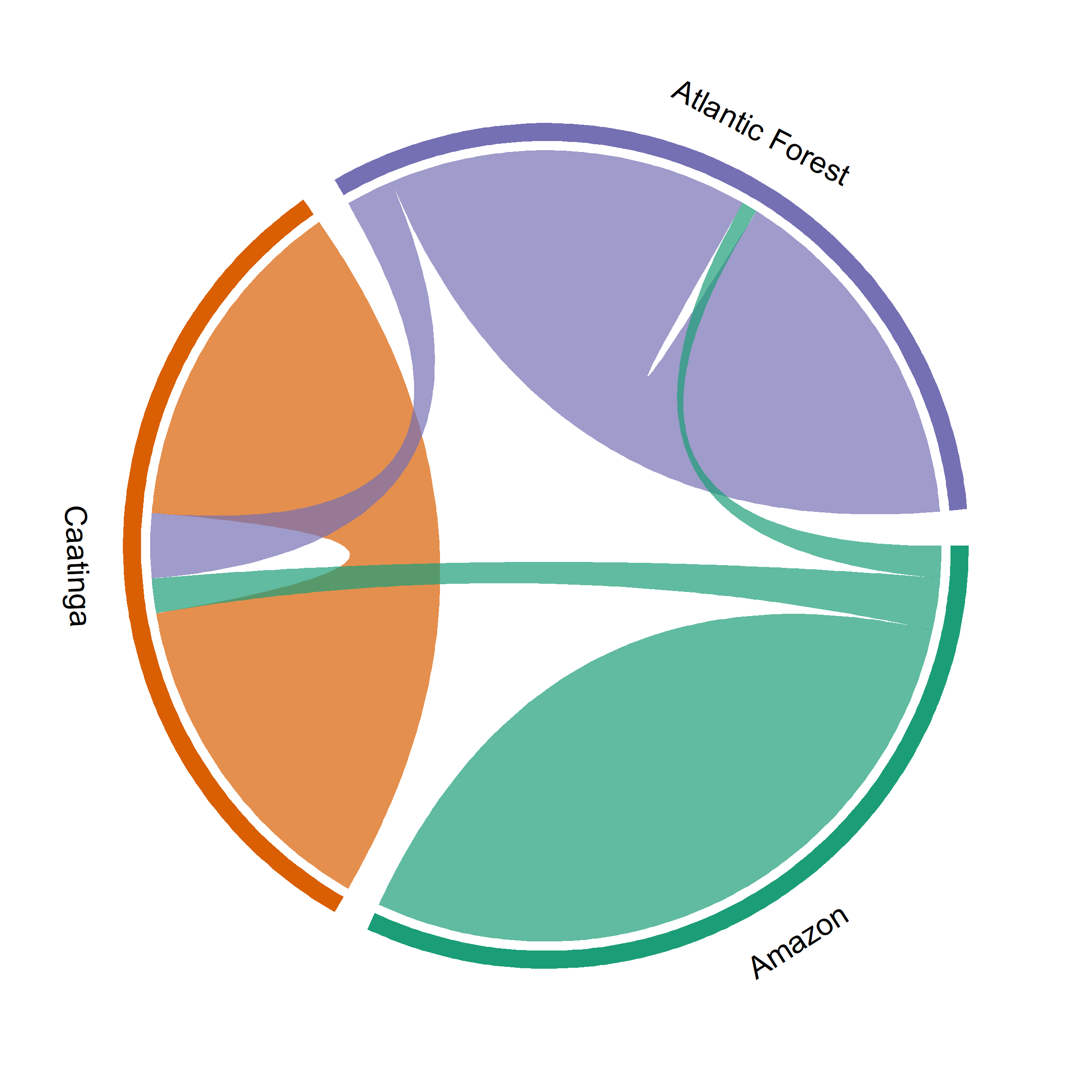


**Estimated Observed**

## *Mapping neotropical inselbergs onto protected areas*

Table 1. Protected areas in which some of the inselbergs analyzed are located, the respective biome in which they are inserted and the code used in the analysis.

| Biome | Protected areas | code |
| --- | --- | --- |
| Amazon | Central Suriname Nature Reserve | SUR-VOL |
| Amazon | Nature Reserve Sipaliwini | SUR-SR |
| Amazon | Parc Amazonien de Guyane | GF-FG |
| Amazon | Parque do Tumucumaque | SUR-SIP |
| Amazon | Parque Estadual do Cristalino | MT-PC |
| Amazon | Parque Nacional Dos Campos Ferruginosos | PA-PHR |
| Amazon | Parque Nacional Natural El Tuparro/ El Tuparro Nature Reserve | CO-CT |
| Amazon | Reserva Forestal Sipapo/ Monumento Natural Piedra La Tortuga | VEN-PTO |
| Amazon | Réserve naturelle nationale des Nouragues | GF-NOU |
| Atlantic Forest | APA da Prainha | RJ-PR |
| Atlantic Forest | APA dos Morros da Babilônia e São João / Parque Natural Municipal Paisagem Carioca | RJ-CH |
| Atlantic Forest | APA Pedra do Elefante | ES-PE |
| Atlantic Forest | APA Waldeir Gonçalves – Serra do Itaóca | RJ-MI |
| Atlantic Forest | Monumento Natural dos Morros do Pão de Açúcar e da Urca | RJ-PA |
| Atlantic Forest | Parque Estadual Da Serra Da Tiririca | RJ-AM |
| Atlantic Forest | Parque Estadual Da Serra Da Tiririca | RJ-IC |
